# Potential Determinants of Cardio-Metabolic Risk among Aboriginal and Torres Strait Islander Children and Adolescents: A Systematic Review

**DOI:** 10.3390/ijerph19159180

**Published:** 2022-07-27

**Authors:** Christopher D. McKay, Eamon O’Bryan, Lina Gubhaju, Bridgette McNamara, Alison J. Gibberd, Peter Azzopardi, Sandra Eades

**Affiliations:** 1 Melbourne School of Population and Global Health, The University of Melbourne, Melbourne, VIC 3010, Australia; lina.gubhaju@unimelb.edu.au (L.G.); bridgette.mcnamara@unimelb.edu.au (B.M.); alison.gibberd@unimelb.edu.au (A.J.G.); sandra.eades@unimelb.edu.au (S.E.); 2 Faculty of Medicine, Dentistry and Health Sciences, The University of Melbourne, Melbourne, VIC 3010, Australia; eamon.obryan@gmail.com (E.O.); peter.azzopardi@burnet.edu.au (P.A.); 3 Global Adolescent Health Group, Maternal, Child and Adolescent Health Program, Burnet Institute, Melbourne, VIC 3004, Australia; 4 St Vincent’s Hospital, Melbourne, VIC 3065, Australia; 5 Murdoch Children’s Research Institute, Melbourne, VIC 3052, Australia

**Keywords:** Indigenous, Aboriginal and Torres Strait Islander, Australia, adolescence, childhood, cardio-metabolic health, metabolic syndrome, obesity, risk factors

## Abstract

Prevention initiatives during childhood and adolescence have great potential to address the health inequities experienced by Aboriginal and Torres Strait Islander (Indigenous) populations in Australia by targeting modifiable risk factors for cardio-metabolic diseases. We aimed to synthesize existing evidence about potential determinants of cardio-metabolic risk markers—obesity, elevated blood pressure, elevated blood glucose, abnormal lipids, or a clustering of these factors known as the metabolic syndrome (MetS)—for Indigenous children and adolescents. We systematically searched six databases for journal articles and three websites for relevant grey literature. Included articles (*n* = 47) reported associations between exposures (or interventions) and one or more of the risk markers among Indigenous participants aged 0–24 years. Data from 18 distinct studies about 41 exposure–outcome associations were synthesized (by outcome: obesity [*n* = 18]; blood pressure [*n* = 9]; glucose, insulin or diabetes [*n* = 4]; lipids [*n* = 5]; and MetS [*n* = 5]). Obesity was associated with each of the other cardio-metabolic risk markers. Larger birth size and higher area-level socioeconomic status were associated with obesity; the latter is opposite to what is observed in the non-Indigenous population. There were major gaps in the evidence for other risk markers, as well as by age group, geography, and exposure type. Screening for risk markers among those with obesity and culturally appropriate obesity prevention initiatives could reduce the burden of cardio-metabolic disease.

## 1. Introduction

Cardiovascular disease (CVD) and type 2 diabetes mellitus (together known as cardio-metabolic diseases) are leading causes of overall disease burden and mortality for Aboriginal and Torres Strait Islander peoples in Australia (hereafter collectively referred to as Indigenous, acknowledging the significant diversity within and between Aboriginal and Torres Strait Islander cultures) [1]. They emerge earlier and at higher rates relative to non-Indigenous Australians [2], consistent with disparities in the social determinants of health [3]. Many major modifiable risk factors for cardio-metabolic diseases, such as obesity, elevated blood glucose, high blood pressure, abnormal blood lipids, and tobacco smoking, are also more prevalent among Indigenous people [2]. Existing evidence indicates that type 2 diabetes mellitus (T2DM) and obesity are increasing among Indigenous children and adolescents, and at higher rates than the non-Indigenous population [4,5,6,7,8,9]. There is huge potential to improve health outcomes by addressing modifiable risk factors in childhood and adolescence [10].

The metabolic syndrome (MetS) refers to a clustering of multiple interrelated cardio-metabolic risk markers that can be reliably measured in clinical settings [11]. As such, it can be used as a diagnostic framework for early detection and treatment of individuals at considerably increased risk of progression to T2DM or CVD, and measurement of these risk markers also commonly occurs in epidemiologic studies [11]. Among adults, those with MetS have double the risk of CVD over the next 5–10 years and a 5-fold increased risk of T2DM compared to those without MetS [12]. The International Diabetes Federation (IDF) consensus definition of MetS in childhood and adolescence requires the presence of abdominal obesity, combined with two or more of the following: elevated triglycerides; reduced high-density lipoprotein cholesterol; raised blood pressure; and elevated blood glucose, or previously diagnosed T2DM [13]. MetS is increasing among children and adolescents globally, in association with the obesity epidemic [13]. A review of MetS prevalence among 2–19-year-olds globally found it ranged from about 2% to 23% in population-based studies, depending on the region and the definition used [14]. It was as high as 60% among overweight and obese young people. Prevalence estimates for Indigenous children and adolescents in Australia are geographically limited and non-representative but are toward the higher end of the global range; 14% among members of a birth cohort in the Darwin region of the Northern Territory [15], and 17% among school children in the Torres Strait Islands [16]. The individual risk marker components of MetS have been shown to persist from childhood and adolescence into adulthood [13]. In one study involving Indigenous young people, having MetS in both childhood and adolescence was associated with subclinical atherosclerosis at 18 years of age, yet the extent of atherosclerosis was reduced for those who only had MetS in childhood [17], illustrating the opportunity to modify outcomes during this stage of life.

The published evidence about potential determinants of MetS among Indigenous children and adolescents in Australia has never been systematically reviewed. Previous related reviews have had a limited focus by outcome or exposure or have not been specific to Indigenous children and adolescents, and few have focused on potential determinants [7,18,19,20,21]. New data from Indigenous cohort studies have since been published that add to the evidence base about cardio-metabolic risk markers. A comprehensive synthesis of this evidence will provide a better understanding of the factors contributing to cardio-metabolic risk during this stage of life, allowing for more targeted prevention measures, and identify where further research is required. It is particularly important to determine the extent to which social and environmental determinants of health have been investigated as these are crucial to understanding health disparities between Indigenous and non-Indigenous populations in Australia and working towards health equity [22,23,24].

This systematic review aimed to: (i) synthesize evidence about factors associated with MetS and its individual cardio-metabolic risk marker components among Indigenous children and adolescents in Australia; (ii) identify the major evidence gaps; and (iii) critically appraise both methodological quality and adherence to Indigenous research values.

## 2. Materials and Methods

The study protocol for this review was registered at the International Prospective Register of Systematic Reviews on 28 April 2020 (PROSPERO, CRD42020166271) and is reported in accordance with the Preferred Reporting Items for Systematic Reviews and Meta-Analyses (PRISMA) [25].

### 2.1. Search Strategy

A search strategy was developed in consultation with a librarian. A systematic search was first conducted on 21 March 2020 on the MEDLINE, EMBASE, PsycINFO, CINAHL, Scopus, and Informit databases, then repeated on 15 December 2021. The search strategy used a combination of subject headings and keywords to identify publications that addressed three search concepts: (i) MetS and its components; (ii) Indigenous populations in Australia; and (iii) the childhood and adolescent period. Supplementary Table S1 presents the search strategy used for MEDLINE, subsequently adapted with the corresponding medical subject headings for the other databases. No restrictions were applied to the search. Relevant websites were also hand searched for grey literature, including the Australian Bureau of Statistics, Australian Institute of Health and Welfare, and Australian Indigenous HealthInfoNet. Reference lists of included articles and relevant reviews were hand searched for any additional studies not captured.

### 2.2. Selection Criteria

Original published research about Indigenous people in Australia aged 0–24 years was included if the article reported data on MetS, or at least one its components, and its relationship with at least one exposure. The IDF definition of MetS was used [13], with the following individual cardio-metabolic risk marker components: obesity; elevated blood pressure; abnormal lipids; and elevated blood glucose or T2DM. Alternative definitions for MetS and alternative measures for the individual components, including body mass index (BMI) and other measures of adiposity, glycated hemoglobin (HbA1c), and insulin and insulin resistance measures, were also included to maximize the capture of relevant information. Studies that focused on participants with type 1 diabetes mellitus (T1DM) were excluded. Studies including adults aged 25 years and over, or non-Indigenous participants, were only included if data were reported separately for Indigenous people under 25 years, or they made up the majority of the sample. Qualitative (and mixed methods) studies were included if they reported data about relevant exposure–outcome relationships. Articles not published in English were excluded, as were conference proceedings, posters or abstracts, editorials, commentaries, perspectives, case studies, review articles, book chapters, and theses.

### 2.3. Screening and Data Extraction

Two reviewers (C.D.M and E.O.) independently screened all article titles and abstracts, then the remaining full text articles, with disagreements resolved through discussion or the adjudication of a third reviewer. The following data were extracted from the included articles: population, setting, design, period, sampling method, sample size, analysis sample and exclusion criteria, age range and/or mean age, outcome and exposure definitions, measure of association and respective confidence intervals or *p*-values. Where a study reported measures of association from multiple iterations of a model, only the results from the final model were extracted.

### 2.4. Quality Appraisal

Methodological quality (risk of bias) was appraised using the National Heart, Lung and Blood Institute (NHLBI) assessment tools relevant to the study design [26]. The tools provide guidance for assigning quality ratings of ‘good’, ‘fair’, or ‘poor’, where good studies have a low risk of bias and poor studies a high risk. Acknowledging that the unique complexities of conducting Indigenous research in Australia often necessitate more pragmatic methods [27,28,29], and because we took a broader view of study quality through the use of a second quality appraisal tool (the Aboriginal and Torres Strait Islander Quality Appraisal Tool [30]), we used the terminology ‘low’, ‘moderate’, and ‘high’ risk of bias instead of the NHLBI terminology. Appraisals were conducted at the article level as individual articles from the same study had used different study designs, data subsets, analysis methods, or focused on different exposure–outcome relationships. In some cases, different exposure–outcome associations from the same article were assigned different ratings due to their different risk of bias (e.g., longitudinal vs. cross-sectional exposure measures). Moderate and high-risk ratings were assigned based on the severity of potential bias, or the cumulative impact of multiple sources of bias, using the NHLBI guidance for determining overall quality. A second reviewer (L.G.) appraised a random 10% sample of the total number of articles (five articles); there was agreement on four out of five articles and the disagreement was resolved through discussion.

The Aboriginal and Torres Strait Islander Quality Appraisal Tool [30], developed by The Centre of Research Excellence in Aboriginal Chronic Disease Knowledge Translation and Exchange (CREATE), was used to assess study quality using a range of questions reflecting Indigenous research values, to account for broader and more culturally appropriate conceptions of research quality [31]. This was conducted at the overall study level, with each study assigned a total score out of 14 based on the sum of component scores awarded according to whether each of the 14 questions could be answered (‘Yes’ [1 point], ‘Partially’ [0.5 points], or ‘No’/‘Unclear’ [0 points]). In scoring the studies, additional information was considered that had been published in referenced study protocols and cohort profile papers, as well as study websites and researcher profiles, where these were available.

### 2.5. Data Synthesis

To conceptualize how exposures at various levels may impact on the cardio-metabolic health of Indigenous children and adolescents, this review drew on bioecological and socioecological models of health [32,33,34]. A simplified framework was used to categorize exposures with the following levels: (i) individual characteristics (e.g., age, sex, biological characteristics, perinatal exposures, and health behaviors); (ii) family/peer health and behaviors (e.g., family diet or physical activity behaviors, and parent health conditions); (iii) social determinants (e.g., income, education, employment, housing, and racism); and (iv) environmental factors (exposures measured at an area level). A qualitative synthesis of extracted data was undertaken with results grouped by outcome, then according to the exposure level framework, and described using the following age subgroups: preschool (0–5 years); childhood (6–14 years); and youth (15–24 years). All quantitative results with low–moderate risk of bias were included in the synthesis. High risk of bias results and null associations were only included where they could be compared with other data about the same exposure with low–moderate risk. An exception was made for the MetS outcome due to the sparsity of data; high-risk data were included in the synthesis if they could be compared with data of any quality about the same exposure. The results from intervention studies and qualitative data are described separately. Due to the inclusion of a wide range of different exposure–outcome relationships, with various outcome definitions and a small number of sources for each relationship, meta-analyses were not possible.

## 3. Results

### 3.1. Search and Study Selection

**Figure 1:**
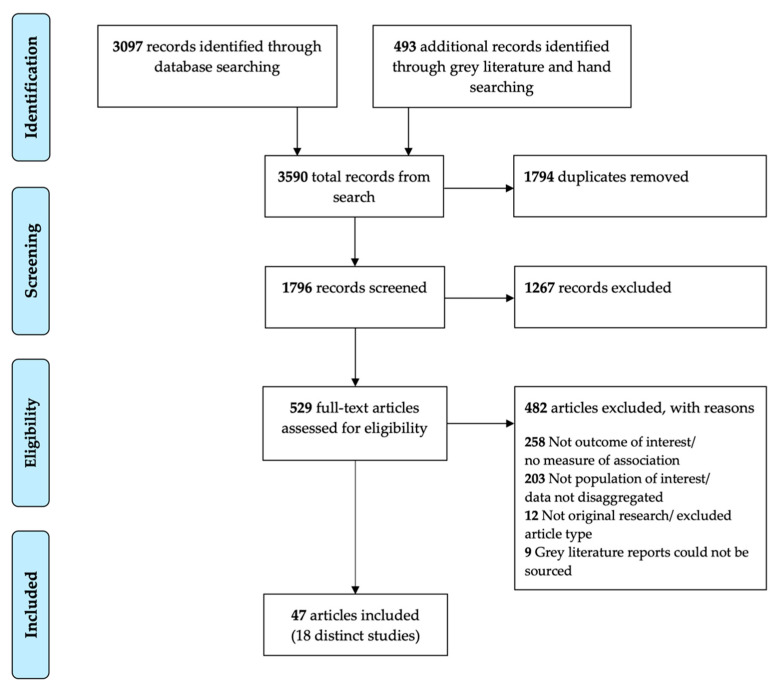
PRISMA flow diagram of study selection.

The search returned 1796 unique records; 1267 were excluded following title and abstract screening. After reviewing the full text of 529 articles, 482 were excluded (Figure 1). There was a very high level of agreement between the two independent reviewers for inclusion and exclusion of full text articles (Cohen’s Kappa 0.87).

### 3.2. Characteristics of Included Studies

Forty-seven articles were included in this review, from 18 distinct studies (Table 1); 44 peer-reviewed journal articles, two grey literature reports, and one non-peer-reviewed article. Twenty-five (53%) articles came from just two longitudinal cohort studies; ‘Aboriginal Birth Cohort’ (ABC, *n* = 16), and ‘Footprints in Time: The Longitudinal Study of Indigenous Children’ (LSIC, *n* = 9). Fifteen of the 18 distinct studies were observational, consisting of longitudinal cohort studies (*n* = 7), cross-sectional studies (*n* = 7), and one mixed methods study. The three intervention studies included a randomized controlled trial (RCT), a pre-post intervention study, and a pre-post intervention study with repeated cross-sections. Most studies included only Indigenous participants within the eligible age range (*n* = 10); or with a disaggregated subgroup, or majority, within the age range (*n* = 5); and three studies included Indigenous and non-Indigenous participants. Although participants of ABC were a mean age of 25 years at the most recent wave of follow-up, we included data from that wave as a one-year age difference was unlikely to meaningfully impact results. Three studies measured outcomes predominately in the preschool age group (0–5 years), six in childhood (6–14 years), five during youth (15–24 years), two spanned both preschool and childhood, and two spanned childhood and youth. One study (LSIC) had a national sample, two articles with an overlapping sample (treated as a single study in this review) recruited from various remote locations around Australia [35,36], and the remainder were from within one state/region (NSW = 6, Queensland = 3, NT = 2, SA = 2, WA = 2, Torres Strait = 1). Obesity measures were the most common outcome of interest and MetS the least (obesity [*n* = 12]; blood pressure [*n* = 9]; glucose, insulin or diabetes [*n* = 6]; lipids [*n* = 5]; and MetS [*n* = 3]).

### 3.3. Study Quality

In respect to methodological quality, five articles reported results with a low risk of bias, 19 had moderate risk, three articles contributed both moderate and high-risk results, and 20 had results with high risk of bias (Supplementary Tables S4–S6). The most common reasons an article was not given a low–risk rating included reliance on cross-sectional data, a potential for selection bias, confounding bias, or measurement error, and insufficient interpretation of how these biases might impact the reported results. One of the most common reasons for high-risk scores was that only unadjusted descriptive data were reported, usually where the main aim of the study was not to measure formal exposure–outcome associations. The mixed methods study was not peer-reviewed and the quantitative results were missing information that would have allowed potential exposure–outcome associations to be interpreted [78], so we were only able to include the qualitative data. An additional source of bias not captured by the NHLBI tool rating is the inclusion of some non-disaggregated data (biased by the inclusion of older adults or non-Indigenous participants), and these instances are noted in the qualitative synthesis.

Scores from the CREATE tool ranged from 0 to 12.5, with 10 of the 18 studies addressing at least half the questions, and four studies had scores of 10 or more (Supplementary Table S7). The criteria most commonly addressed were: ‘Was community consultation and engagement appropriately inclusive?’ (14/18 Yes); and ‘Did the research have Aboriginal and Torres Strait Islander governance?’ (13/18 Yes). Only one study provided information addressing the two criteria related to intellectual and cultural property rights, while only two studies had clearly used an Indigenous research paradigm and strengths-based approaches to research. Most studies (17/18) provided insufficient information to appraise all criteria.

### 3.4. Summary of Exposures Associated with Cardio-Metabolic Risk Markers

Following the quality appraisal, quantitative data about 41 exposure–outcome associations from 17 studies (43 articles) and qualitative data from one study (one article) were included in the qualitative data synthesis. Results from three articles are not described further in the qualitative synthesis as they had a high risk of bias and there was no comparable data from other studies [17,36,62] (see Supplementary Table S2). Overall, 24 exposures (or interventions) were identified (Table 2). Eighteen exposures were investigated in relation to obesity and fewer associations with the other outcomes were reported (blood pressure [*n* = 9]; glucose, insulin or diabetes [*n* = 4]; lipids [*n* = 5]; and MetS [*n* = 5]). By the exposure level framework, 14 exposures were individual characteristics, with limited data for other levels (family/peer [*n* = 1]; social determinants [*n* = 5]; and environmental factors [*n* = 2]). Higher obesity measures (e.g., higher BMI or waist circumference) were associated with elevated blood pressure, abnormal lipids, and elevated glucose, insulin or diabetes outcomes in multiple studies and no conflicting results were found. Larger birth size and higher area-level socioeconomic status (SES) were each associated with higher obesity outcomes in three studies. Among the findings from single studies, there was evidence of an association with obesity (or elevated blood pressure) for three maternal factors (maternal obesity, lower parity and higher maternal age), and for three behavioral factors (lower physical activity, lower sleep duration, and poorer diet). Aside from area-level SES, there was insufficient evidence to draw overall conclusions from exposures at the social and environmental level. A full list of the null associations is presented in Supplementary Table S3.

### 3.5. Associations with MetS and Cardio-Metabolic Risk Marker Clustering

Results from three studies (five articles) that investigated associations with MetS or other measures of risk marker clustering were included in the synthesis, covering five exposures, with participants aged 8–28 years (Table 3). Each article used a different outcome definition, including four variations on the MetS definition. Two alternative outcomes included were: ‘adverse cardio-metabolic profile’, which included C-reactive protein as a risk marker, in addition to the traditional MetS components, and required a clustering of three or more factors [48]; and ‘ideal cardiovascular health score’, which was a score ranging 0–7 with one point given for being in the ideal range for BMI, blood pressure, total cholesterol, HbA1c, physical activity, diet, and non-smoking [46].

#### 3.5.1. Individual Characteristics

**Age (two studies)**. There was no difference in mean age between children (aged 8–14 years) with and without MetS in ABC [15], while in another study the proportion of MetS among 20–25-year-old youth was double that of 15–19-year-olds (19.8 vs. 9.7%) [65].

**Sex (three studies)**. There were conflicting results for differences in MetS and cardiovascular health score by sex, with females in two studies at greater risk compared to males during childhood and youth [16,46] and the third indicating that males were at higher risk during youth only [65].

**Obesity measures (one study)**. In ABC, higher BMI and alternative obesity measures were associated with a higher number of abnormal cardio-metabolic markers or MetS in childhood and youth [15,48].

#### 3.5.2. Social Determinants

**Individual socioeconomic factors (one study)**. Among females in a majority Indigenous (80%) sample from ABC, odds of adverse cardio-metabolic profile when participants were aged 25 years were 72% lower for those who owned a car compared to those with no car in the household [48]. An opposite association was observed for males, but was not statistically significant, and no data were available for males and females combined. There was no association with years of schooling or main source of household income.

#### 3.5.3. Environmental Factors

**Remoteness (one study)**. In ABC, females living in remote areas had 10-fold higher odds of adverse cardio-metabolic profile at 25 years of age compared to females in urban areas [48]. An opposite association was observed for males, but was not statistically significant, and no data were available for males and females combined. There was no association between remoteness and MetS at 11 years (high risk of bias) [15].

### 3.6. Associations with Obesity Outcomes

Results from ten studies (26 articles) that investigated associations with obesity measures were included in the synthesis, covering 17 exposures and three interventions, with participants aged 1–28 years (Table 4). Fourteen different types of obesity outcome measure were included: BMI, BMI z-scores (zBMI), BMI categories (overweight, obese, ideal BMI), rate of BMI change, waist circumference (WC), WC z-score (zWC), elevated WC, elevated waist-height ratio (WHtR), waist-hip ratio (WHpR), total body fat percentage, mid-upper arm circumference, subscapular skinfold, triceps skinfold, and subscapular-triceps skinfolds ratio.

#### 3.6.1. Individual Characteristics

**Sex (three studies)**. For BMI measures, there was no clear overall trend by sex in any age subgroup [46,49,52,53,58,63]. In ABC, elevated WHtR was more common among females at 11 and 18 years of age (high risk of bias) [49].

**Birth size (four studies)**. Three studies (mixed quality ratings) found that small birth size (low birth weight or fetal growth-restricted) was associated with reduced likelihood of obesity and/or lower obesity measures across all three age subgroups [39,41,49,50,52,58,64]. For example, in LSIC a child born large for gestational age (with a birthweight z-score of +1.28) would be predicted to have a BMI z-score 0.56 units higher than a child born small for gestational age (with a birthweight z- score of −1.28), and 0.28 units higher than a child born at the median [52]. No association was seen in the fourth study (high risk of bias) [63].

**Maternal obesity measures (three studies)**. When considering data from ABC and LSIC together, higher maternal weight or weight gain during pregnancy appears to be associated with higher BMI in childhood and youth [46,49,52], though wide confidence intervals or estimates with high risk of bias mean there is substantial uncertainty in this finding. No association between maternal body fat and obesity measures was observed among preschool aged children in a third study (high risk of bias) [64].

**Maternal smoking in pregnancy (two studies)**. In LSIC, there were mixed results for the association between maternal smoking and BMI depending on the study wave [52,58], and no association in a second study (high risk of bias) [64].

**Other maternal factors (two studies).** In LSIC, higher maternal age was associated with higher BMI in preschool and childhood [58] and in ABC, higher maternal parity was associated with lower BMI during youth [46,47].

**Diet(two studies)**. Higher consumption of high-fat foods (≥2 occasions the previous day vs. <2) was associated with a greater increase in BMI over 4 years of childhood in LSIC [53]. Higher sugar-sweetened beverage (SSB) consumption (vs. lower) was associated with higher mean BMI, and a greater increase in BMI over 4 years, though the latter was only statistically significant in a sensitivity analysis that included fruit juice in the definition of SSB [53]. Among Torres Strait Islander children and youth, higher (≥2 times per week) dugong consumption (a commonly consumed local food with high energy content when fried) was associated with 1.9-fold increased odds of overweight/obese compared with lower consumption, yet no association was observed by level of consumption of vegetables, fruit, takeaway food, fish, or turtle [67].

**Sleep (one study)**. Shorter sleep duration and later bedtime were associated with higher mean BMI and greater increase in BMI over four years of childhood in LSIC [55,59].

**Physical activity (one study)**. Fewer days of physical activity (0–3 vs. 4–7) in the previous week was associated with 2.5-fold increased odds of overweight/obese and 2.9-fold increased odds of elevated WC among Torres Strait Islander children and youth [67].

#### 3.6.2. Social Determinants

**Racism (two studies)**. In LSIC, longitudinal data indicate that caregiver-reported exposure to racism during preschool and earlier childhood ages was associated with obesity at two follow-up points in later childhood (5–10 and 7–12 years of age), with estimates in the range of a 1.6- to 1.7-fold increase in odds for those exposed compared to non-exposed [54,56]. However, cross-sectional data from ABC (high risk of bias) did not show an association between self-reported racism exposure and BMI or WHpR at 18 years of age [42].

**Family socioeconomic factors (two studies)**. Longitudinal data from the first four waves of LSIC indicate that maternal education below Year 12 is associated with higher BMI during preschool and childhood, though no association was observed with mother’s employment status [58]. No association was observed between maternal education and overweight/obesity in childhood in a second study (high risk of bias) [63].

**Other social determinants (two studies)**. Higher cultural-based resilience among participants’ mothers was associated with higher BMI during childhood in LSIC [58]. In another study involving a custodial sample, data disaggregated for Indigenous participants indicated 6.9-fold higher odds of overweight/obese during youth for those incarcerated for 12 months or longer compared to shorter periods [69].

#### 3.6.3. Environmental Factors

**Area-level SES (four studies)**. Overall, greater area-level SES was associated with higher obesity measures. In ABC and LSIC, higher BMI or levels of obesity were consistently seen among children and youth who were living in more advantaged areas at baseline [46,47,49,52,53,56]. For example, LSIC participants from areas with highest disadvantage had 80% lower odds of obesity in childhood compared to the least disadvantaged areas [56], and ABC participants from least disadvantaged areas had 91% lower odds of ideal BMI at 25 years of age compared to most disadvantaged areas [46]. Results from a cross-sectional study of preschool children were also consistent with this (high risk of bias) [68]. The one study where no association was observed had a small sample and high risk of bias [63].

**Remoteness (three studies)**. There were conflicting findings on remoteness and obesity measures. In ABC, higher obesity measures during childhood and youth were consistently found among those living in urban areas compared to remote [37,47,49]. However, remote children had higher subscapular/triceps skinfolds ratio in childhood, indicating a body fat distribution with greater truncal fat [37]. No consistent association was observed between remoteness and BMI during childhood in LSIC [51,53,55,58]. No association with BMI was observed in a cross-sectional study of preschool children (high risk of bias), though it was noted some remote populations were not included in the study [68].

#### 3.6.4. Interventions (Three Studies)

Reductions in obesity measures were not found in any of the three intervention studies, which all targeted health behavior change. The highest quality data came from the Baby Teeth Talk RCT which involved a culturally appropriate, multi-faceted oral health intervention for mothers of Indigenous babies [74,75]. At two years of age, there was no difference in obesity measures between the treatment and control group [74]. At three years of age, when both groups had received the intervention, BMI and arm circumference measures were higher among the group that received the intervention first, compared to the group with delayed access [75]. The two pre-post studies, which focused on family fruit and vegetable intake [76], and nutrition and physical activity at the school and community level [77], found no changes in obesity measures among children and youth (high risk of bias).

#### 3.6.5. Qualitative Data (One Study)

Interviews with mothers and grandmothers showed that perceptions about the determinants of childhood obesity spanned individual characteristics to environmental factors [78]. Islander ethnicity was identified as being associated with bigger body size, and lack of exercise, sedentary lifestyles and food portion sizes were perceived as contributing to obesity. Diet and physical activity were linked to social and environmental factors, as follows: lack of specific education for parents about appropriate foods and weight management; food security and economic issues mean families often opt for less healthy but cheaper and more filling foods, particularly when catering for large families; high costs of gyms and organized sports; and the ubiquity of fast-food restaurants, which are attractive to children.

### 3.7. Associations with Blood Pressure Outcomes

Results from five studies (14 articles) that investigated associations with blood pressure were included in the synthesis, covering nine exposures and one intervention, with participants aged 2–29 years (Table 5). Three types of outcome measures were used: continuous systolic and diastolic blood pressure measures (SBP and DBP); age, sex and height-specific SBP and DBP z-scores (zSBP and zDBP); and categories such as elevated blood pressure/hypertension, and ideal blood pressure.

#### 3.7.1. Individual Characteristics

**Sex (three studies)**. Males had higher blood pressure than females in the two studies with youth populations (mixed quality) [44,46,70], and in the third study with participants predominately in childhood there was no difference by sex [60]. In ABC, females had 5.5-fold higher odds of ideal blood pressure than males at 25 years of age [46], and in a cross-sectional study (high risk of bias), male youths had 4.4-fold higher odds of hypertension than females [70].

**Obesity measures (three studies)**. There was a positive relationship between BMI and blood pressure in all three studies. This association was observed at all three follow-ups of ABC, from 11 to 25 years of age [44,45,48]. BMI at 18 years, which was strongly predicted by BMI at 11 years, was the variable with the largest effect on SBP and DBP at 18 years, and a pathway analysis indicated all other indirect effects (sex, birth weight, smoking and remoteness) were mediated through BMI [44]. Two cross-sectional studies found similar associations: a positive linear relationship between age and sex-specific BMI and blood pressure z-scores for children [60]; and 4.6-fold higher odds of hypertension for youth with obesity compared to normal weight (high risk of bias) [70].

**Kidney size (one study)**. Kidney length and volume were negatively associated with SBP among children [72].

**Birth size (two studies).** Overall, there was no consistent evidence of an independent association between birth weight and blood pressure, though some evidence of a mediated effect through later BMI. Across three waves of follow-up in ABC, there was no consistent association after adjusting for contemporary BMI [40,44,45,46,50]. A pathway analysis indicated that small positive effects of birth weight on blood pressure were entirely mediated through BMI [44]. In a cross-sectional study, there was no association between birth weight and blood pressure among children and youth, before or after adjusting for current weight [71].

**Maternal obesity measures (one study)**. In ABC, those born to obese mothers had 87% lower odds of ideal blood pressure at 25 years than children of normal weight mothers [46].

**Maternal smoking (two studies)**. In ABC and a cross-sectional study, there was no association between maternal smoking during pregnancy and blood pressure among children and youth [44,60].

#### 3.7.2. Family/Peer Health and Behaviors

**Caregiver blood pressure (one study)**. A positive cross-sectional association was observed between caregiver SBP and child blood pressure [60].

#### 3.7.3. Environmental Factors

**Area-level SES (one study)**. In ABC, there was a trend for higher blood pressure with increasing area-level SES at 18 and 25 years [47], with those from the least disadvantaged areas having 95% lower odds of ideal blood pressure at 25 years compared to the most disadvantaged [46].

**Remoteness (one study)**. In ABC, there was evidence of higher blood pressure in urban compared to remote areas at all three follow-up waves [37,44,46].

#### 3.7.4. Interventions (one study)

In the Baby Teeth Talk RCT, there was no difference in blood pressure between the oral health intervention group and control group at 2 years of age [74], and at three years of age there was no difference between the group who received the intervention first and the group with delayed access [75].

### 3.8. Associations with Glucose, Insulin and Diabetes

Results from five studies (11 articles) that investigated associations with blood glucose, insulin or diabetes outcomes (collectively ‘metabolic outcomes’) were included in the synthesis, covering four exposures, with participants aged 5–34 years (Table 6). There were four types of outcome measures: continuous measures of blood glucose, insulin and HbA1c; glucose and insulin tertiles; Homeostasis Model Assessment of Insulin Resistance (HOMA-IR) scores; and the binary categories of elevated glucose, elevated HbA1c, ideal HbA1c, impaired glucose tolerance (IGT), and diabetes/T2DM.

#### 3.8.1. Individual Characteristics

**Sex (three studies)**. In two studies, no association was observed between sex and HbA1c levels during childhood and youth [46,61]. In a third study (high risk of bias), females had higher fasting and 2-h (post oral glucose tolerance test) insulin levels during youth [66].

**Obesity measures (five studies)**. In studies including children and youth, there were consistent positive relationships between obesity measures and metabolic outcomes, though most had a high risk of bias. In ABC and one other longitudinal study (high risk of bias), there were positive associations between BMI and each of insulin (fasting and 2-h), HOMA-IR score, fasting glucose, elevated HbA1c and abnormal glucose tolerance (IGT or diabetes) during childhood and youth [15,40,43,48,66], with ABC also reporting a relationship between WC and HOMA-IR [15]. In one cross-sectional study (high risk of bias), there was a 2.5-fold (95% CI: 0.73, 8.63) higher prevalence of elevated HbA1c among obese compared to normal weight children and youth [61], and in another (high risk of bias) there were positive relationships between each of BMI, WC and overweight/obese with HOMA-IR and fasting insulin [16]. Results from a third cross-sectional study (high risk of bias), disaggregated for 15–34-year-olds from an adult sample [35], showed higher odds of IGT and T2DM at higher BMI categories, in agreement with Braun et al. (1996).

**Birth size (one study)**. In ABC, no associations were observed between birth size and glucose or insulin levels at 11 years [38,40]. There was a weak positive association between birth weight and fasting glucose levels at 18 years, with fetal growth restriction associated with lower glucose, before and after accounting for contemporary height and weight [43]. Further analysis of the relative contributions to total variance found that for fasting glucose levels, the effect of birth weight and current weight was similar (R^2^ 0.070 vs. 0.076), and for fasting insulin levels, the effect of current weight was considerably stronger than birth weight (R^2^ 0.299 vs. 0.019). There was no association between birth weight and ideal HbA1c at 25 years [46].

#### 3.8.2. Environmental Factors

**Remoteness (one study)**. In ABC, children living in urban areas at 11 years of age had higher median fasting insulin than remote children, though no association was seen with fasting glucose [37]. At 25 years of age, there was no association between mother’s remoteness at birth and ideal HbA1c [46].

### 3.9. Associations with Lipid Outcomes

Results from four studies (10 articles) that investigated associations with lipid outcomes were included in the synthesis, covering five exposures, with participants aged 8–29 years (Table 7). The outcomes were either continuous measures or dichotomized by risk level and included: total cholesterol; high-density lipoprotein cholesterol (HDL-c); low-density lipoprotein cholesterol (LDL-c); and triglycerides (TG).

#### 3.9.1. Individual Characteristics

**Sex (two studies)**. In a cross-sectional study, females aged 5–18 years had 54% higher prevalence of low HDL-c than males [61], though it was not statistically significant (95% CI: 0.97, 2.47). In ABC, there was no association between sex and ideal total cholesterol at 25 years [46].

**Obesity measures (four studies)**. There was consistent evidence of an association between obesity and abnormal lipids. Longitudinal data from ABC show that BMI in childhood (11 years) and youth (18 years) was positively associated with total cholesterol levels at 18 years [45]. Consistent evidence was found in three cross-sectional studies (high risk of bias) [16,61,73]. In three studies (mixed quality), there was cross-sectional evidence that higher BMI was associated with lower HDL-c [16,45,48,61]. For example, in one study obese children and youth had double the prevalence of low HDL-c compared to those with normal weight [61]. Two of the four studies measured TG, with a positive association with BMI in each, though both had a high risk of bias [16,48].

**Birth size (one study)**. There was no clear association between birth weight and lipid outcomes. For ABC participants aged 11 years, there were higher levels of TG with increasing birth weight for gestational age category, though the association did not persist after adjusting for contemporary BMI, indicating that any effect of birth weight is likely mediated through later BMI [50]. At the same age, continuous birth weight (kg) was not clearly associated with any of the lipid outcomes [40].

**Maternal obesity measures (one study)**. ABC participants born to obese mothers had 87% lower odds of ideal total cholesterol at 25 years, compared to children of normal weight mothers [46].

#### 3.9.2. Environmental Factors

**Area-level SES (one study)**. The association between area-level SES and abnormal lipids was unclear in ABC. There was a trend for lower levels of HDL-c (a negative outcome) across all three waves among those whose mothers were living in higher disadvantage areas at time of birth, yet higher disadvantage was also associated with lower levels of LDL-c (a positive outcome) in the last two follow-ups [47], and there was no association with ideal total cholesterol at 25 years [46].

**Remoteness (one study)**. In ABC, higher HDL-c and lower TG were observed at all three waves among those with mothers living in urban areas at time of birth, compared to remote [47], and there was a cross-sectional association between living in an urban area at 11 years and higher total cholesterol and HDL-c [37]. However, remoteness at birth was not associated with ideal total cholesterol at 25 years [46].

## 4. Discussion

To our knowledge, this is the first comprehensive review of potential determinants of MetS and its individual cardio-metabolic risk marker components among Indigenous children and adolescents in Australia. The current review presents evidence from multiple sources showing obesity is strongly associated with each of the other cardio-metabolic risk markers, indicating that it plays an important role in cardio-metabolic risk among young Indigenous populations. Larger size at birth is associated with higher obesity measures across the childhood and adolescent age spectrum, with limited evidence that maternal factors and child health behaviors are also associated with child obesity and elevated blood pressure. While these findings are consistent with what is seen in other populations [79,80,81], at the environmental level, evidence from multiple studies included in this review indicates that higher area-level SES is strongly associated with higher levels of obesity among Indigenous children and adolescents. This is opposite to what is seen in the general Australian population [82,83], highlighting the need for more within-population research on the social determinants of cardio-metabolic health to understand and address the root causes of health inequities for Indigenous populations. Evidence for predictors of MetS and the other cardio-metabolic risk markers is largely lacking. This review identifies major gaps in the evidence base about behavioral exposures, family/peer factors and social determinants, as well as by age group and geography.

Obesity is strongly associated with other cardio-metabolic risk markers in childhood and adolescence. There was evidence from multiple sources showing that elevated obesity measures were associated with each of elevated blood pressure; elevated glucose, insulin, or diabetes; and abnormal lipids. Longitudinal evidence also indicates that obesity in childhood is associated with obesity in adolescence, and the same was seen for MetS [17]. The review findings suggest that obesity is likely to play an important role in the early emergence of cardio-metabolic disease among Indigenous populations. Current medical consensus in Australia recommends screening for cardiovascular risk factors among Indigenous adults from 18 years of age to identify those at risk of a primary CVD event [84]. Our findings, and other evidence about subclinical CVD [17], indicate that guidance is also needed about screening adolescents with obesity to reduce their risk of future CVD. The findings support expert recommendations that Indigenous children and adolescents aged over 10 years in rural and remote areas be screened for T2DM if they have obesity, and that more effective prevention strategies are needed for this population that take into account social determinants of health and societal level factors [85]. However, this guidance should be extended to adolescents in urban areas also.

Larger birth size is associated with elevated obesity measures. There is strong evidence from multiple sources for this association during childhood and adolescence, though there is not consistent evidence of associations with other risk markers. This finding also reflects the association of low birth weight with lower BMI. A previous review that investigated the effect of early life factors on later life cardio-metabolic diseases among Indigenous populations (including adults) from Australia, Canada, New Zealand and USA, observed positive associations between birth weight and later life obesity measures in some Indigenous populations [20], and this association has also been reported in other populations by international reviews and meta-analyses [80,81]. We also found limited evidence that higher maternal age, lower parity and higher weight during pregnancy may be associated with child obesity or elevated blood pressure. Future follow-up of the LSIC cohort, and other birth cohorts involving Indigenous participants, could help confirm the persistence of associations between perinatal factors and obesity into later life, as have been reported in ABC. As higher maternal BMI is associated with larger birth weight babies, and Indigenous mothers give birth at younger ages on average, preventing obesity during adolescence could have intergenerational health benefits for Indigenous populations [86].

Higher area-level SES (less disadvantage) is associated with obesity in childhood and adolescence. This finding conflicts with what has been reported in the general Australian population [82,83], and among Indigenous adults [87], where increased risk of obesity is associated with lower area-level SES. Although these area-level measures are used as a proxy for individual-level SES, there is a lack of published data to confirm individual-level associations among Indigenous children and adolescents, which could help explain this important difference with the non-Indigenous population. It is probable that area-level SES reflects additional environmental factors, such as those identified in the qualitative data included in this review, and by other research into the barriers to healthy nutrition and physical activity among Indigenous young people: lack of access to healthy foods, low cost sports activities, outdoor play spaces, and transport; the abundance of fast-food outlets; and neighborhood safety concerns [78,88,89]. Generally, these environmental factors contribute to poorer health outcomes in low SES areas, yet the results of this review indicate a more complex situation for Indigenous young people and obesity. High-quality evidence included in this review indicated that poor diet was associated with elevated BMI in childhood [53], and this is likely to be most effectively addressed by improving food environments [90], yet a better understanding of the individual and environmental factors behind the differences in obesity by area-level SES will help ensure health interventions are culturally appropriate and successful for the Indigenous population. Future research should aim to tease apart the roles of these factors and explain why the relationship between area-level SES and obesity differs for Indigenous young people.

There are major research gaps about potentially important determinants of cardio-metabolic risk markers. The three studies that had measured MetS or cardio-metabolic risk marker clustering were geographically limited samples from northern Australia, therefore excluding the most populated regions of Australia. The only outcome with reasonable geographic and age group coverage overall was obesity, and Indigenous populations in Tasmania and Australian Capital Territory were not represented at all. Two previous reviews of the extent and quality of health research about Indigenous young people found that urban populations were underrepresented [91,92], and this is still the case. In terms of potential determinants, even for obesity—an important and relatively well-researched health outcome—evidence about known determinants such as diet and physical activity was from samples limited by geography or age group. There are no data on associations between behavioral factors and obesity for youth populations (15–24 years) outside the Torres Strait, and no longitudinal data. Similarly, there are almost no data on the role of family or peer health and behaviors, which are known to have a strong influence on child and adolescent health [33], with peer influences becoming increasingly important during adolescence. Cultural factors and racism are likely to be important health determinants for Indigenous populations [93,94], yet there is insufficient evidence to draw conclusions about their relationship to cardio-metabolic risk. LSIC findings indicated that racism is associated with obesity in childhood, yet no association was found for ABC participants at 18 years. This conflict may reflect the different exposure measures used (carer-reported vs. self-reported), the different age groups (childhood vs. youth) or geographical differences (predominately remote NT vs. diverse national sample). These areas deserve further investigation, using consistent and validated exposure measures. A recent study with a national sample of Indigenous adults, using a validated discrimination measure, found that those who had experienced moderate–high racial discrimination had increased prevalence of high blood pressure (24% higher), high cholesterol (17% higher), diabetes (57% higher) and heart disease (52% higher), than those with no discrimination [95]. Evidence about other potential social determinants during childhood and adolescence is largely lacking and a comprehensive investigation of these factors is required so that the underlying drivers of inequities in cardio-metabolic disease for Indigenous people can be addressed.

Evidence about the effectiveness of interventions in reducing cardio-metabolic risk markers is very limited. Except for the single RCT, the study designs and approaches to analysis were not adequate to distinguish the effects of the intervention from potential confounding factors [96]. The small number of interventions that were identified by this review reflects problems with engagement that have been highlighted previously: Indigenous engagement in existing Australian childhood obesity prevention programs is unknown in many cases and where it has been assessed, engagement has been found to be lower than for the broader population [97]. This reinforces the need for Indigenous governance and leadership in the development of Indigenous-specific obesity programs and the empowerment of Indigenous communities to control such programs [97]. Our review also highlights that there is a lack of good quality data from observational studies within Indigenous populations that could inform the design of interventions.

The quality appraisals reveal opportunities for improvement in future research. For the CREATE tool, most studies had reported on criteria that are likely to take part in the development stage of a study, such as community consultation and engagement and establishing Indigenous governance structures. There appears to be improvement, at least within the scope of this review, since a 2009 review of Indigenous child health studies found fewer than 30% of included studies reported Indigenous involvement [92]. Fewer studies had provided information about Indigenous control over data collection, respect for community protocols, policy impacts, or benefits and capacity building for Indigenous people. This may reflect poorer research practice in latter stages of studies, the priorities of ethics and grant committees, or difficulties including this type of information in journal articles. Only a few studies had used Indigenous research paradigms, strengths-based approaches, or had reported on processes for protecting intellectual and cultural property of Indigenous participants. The absence of definitive information in many articles makes overall conclusions about quality difficult. However, as the CREATE tool and other criteria for reporting on Indigenous research have only recently been published [98], they may help drive improvements in design and reporting into the future.

In respect to methodological quality, many of the most common risk of bias issues identified are things that can be improved upon in the analysis phase with more robust methods [99,100,101], meaning that better quality evidence could potentially be obtained from existing data. Most evidence with low–moderate risk of bias came from ABC and LSIC, illustrating the benefits of investing in Indigenous longitudinal studies. Results from future waves of LSIC, together with forthcoming results from studies that have measured multiple cardio-metabolic risk markers among Indigenous children and adolescents [102,103], will likely address some of the evidence gaps identified and complement findings from this review.

The strengths of this review include the systematic approach to searching multiple relevant databases and grey literature sources; synthesizing evidence for multiple important cardio-metabolic risk markers in order to identify potential determinants from sparse data; interpretation of results using an exposure-level framework and age subgroups to reveal important evidence gaps; and a broader, culturally appropriate approach to quality appraisal that includes methodological quality and Indigenous research values. The major limitations include the limited availability of consistently measured data for specific exposure–outcome relationships; inclusion of multiple study designs; and inclusion of some data not disaggregated for the population of interest. Although we did not include T1DM as an outcome, most of the studies that investigated glucose, insulin, diabetes or MetS outcomes did not report whether they had accounted for potential T1DM cases in their study sample. Considering that the rates of T1DM and T2DM are similar in Indigenous children and adolescents [6], it is possible that T1DM cases were included in those studies and may have biased associations. Modified MetS criteria have been recommended for individuals with T1DM [104], which future studies should consider. However, as young people with T1DM have lower BMI on average than those with T2DM [6], our finding that obesity is associated with elevated glucose, insulin and diabetes is unlikely to be impacted by this potential bias. Finally, the limitations related to limited availability of data were expected and informed the broad research questions and less restrictive approach to study inclusion. In the context of this review, the overall approach might be considered a strength in allowing the substantial research gaps to be identified.

## 5. Conclusions

This review of the potential determinants of MetS among Indigenous children and adolescents indicates that obesity is strongly associated with other cardio-metabolic risk markers. Indigenous adolescents with obesity should be screened for additional cardio-metabolic risk markers to help prevent T2DM and CVD. Culturally appropriate, community-led obesity prevention initiatives that promote healthy behaviors among children and adolescents are also likely to help lower the risk of cardio-metabolic disease in the population. Findings indicate that improvements in Indigenous maternal and perinatal health could improve the cardio-metabolic health of children in later life and deliver intergenerational benefits. Further longitudinal studies are needed that cover the diversity of Indigenous populations and the adolescent age period, that consider social and environmental determinants of cardio-metabolic health, and allow for the effects of environmental factors to be distinguished from individual-level factors. Future research investment should also follow principles for achieving health equity for Indigenous young people [105], and support and empower Indigenous populations to have greater control over their own health.

## Figures and Tables

**Table 1:** Summary of included studies and articles.

Articles (First Author and Year); (Study Name) ^1^	State/ Region; Sampling ^2^	Population Description; Sample Size ^3^	Study Period; Age Range (and/or Mean) ^4^	Outcomes ^5^				
				Obesity	BP	Glucose	Lipids	MetS
**Longitudinal Studies**								
Mackerras 2003 [37], Sayers 2004 [38], Sayers 2007 [39], Sellers 2008 [15], Sayers 2009 [40], Sayers 2011 [41], Priest 2011 [42], Sayers 2013 [43], Mann 2015 [44], Juonala 2016 [17], Gialamas 2018 [45], Sjöholm 2018 [46], Juonala 2019 [47], Sevoyan 2019 ^†^ [48], Sjöholm 2020 [49], Sjöholm 2021 [50]; (ABC)	NT; Hospital	Singletons delivered at Royal Darwin Hospital between January 1987 and March 1990 to an Aboriginal mother; *n* = 686	1987–2016; W2: 8–14 (11), W3: 16–20 (18), W4: 23–28 (25)	X	X	X	X	X
Thurber 2013 ^‡^ [51], Thurber 2015 [52], Thurber 2017 [53], Shepherd 2017 [54], Deacon-Crouch 2018 [55], Cave 2019a, [56] Cave 2019b [57], Westrupp 2019 [58], Fatima 2020 [59]; (LSIC)	National; Community/ population database	Indigenous children aged 0.5–2 years (‘younger cohort’) and 3.5–5 years (‘older cohort’) at baseline (2008), purposively recruited using administrative databases and local community networks at 11 undisclosed sites representing a mix of remote, regional and urban Australian locations; *n* = 1759	2008–2015; W1: 0.5–5, W3: 2–7, W4: 3–9, W6: 5–10, W7: 6–12, W8: 7–12 (9)	X				
Larkins 2017 [60], Riley 2021 [61]; (SEARCH)	NSW; Clinic/community	Aboriginal children aged 0–17 years (with a parent/ caregiver >16 years) who attended one of four participating ACCHS in urban and large regional centers in NSW (Mount Druitt, Campbelltown, Wagga Wagga, Newcastle), recruited between 2008 and 2011; *n* = 1594	2008–2020; Baseline: 2–17 (6.3), W2: 5–18		X	X	X	
Webster 2013 [62], Denney-Wilson 2020 [63]; (Gudaga study)	NSW; Hospital	Aboriginal infants born at Campbelltown hospital or to mothers who resided in the Campbelltown region of Sydney between October 2005 and May 2007; *n* = 159	2007–2016; W1: 2, W2: 9	X				
Pringle 2019 [64]; (Gomeroi gaaynggal study)	NSW; Clinic	Infants born since 2010 to mothers who identified during pregnancy as Indigenous or who were carrying an Indigenous infant and attended a participating antenatal clinic or AMS in Tamworth or Walgett, NSW; *n* = 245	2010–2017; 1–3 (2)	X				
Campbell 2019 [65]	Qld.; Clinic	Indigenous people aged 15–25 years who attended Gurriny Yealamucka Health Service Aboriginal Corporation in Yarrabah between 2013 and 2016 for a Young Persons Check (Medicare item 715); *n* = 433	2013–2016 ^; 15–25	X	X	X	X	X
Braun 1996 [66]	WA; Random/community	A random, opportunistic sample of 100 apparently healthy Aboriginal people aged 7–18 years in 1989, with 25 each from 4 communities in the Kimberley region of WA, followed up after 5 years; *n* = 100	1989–1994; Baseline: 7–18 (13), Follow-up: ~12–23 (18)			X		
**Cross-Sectional Studies**								
Valery 2009 [16], Valery 2012 [67]	Torres Strait; School	Students aged 5–17 years who attended one of the five schools across four Torres Strait islands: Thursday Island, Horn Island, Sue Island, and Mabuiag Island; *n* = 327	2003; 5–17 (11.2)	X	X	X	X	X
Spurrier 2012 [68]	SA; Preschool	Children attending preschool or kindergarten in SA in 2009 aged 3–6 years; *n* = 337 ^§^	2009; 3.5–6 (4.8)	X				
Haysom 2013 [69]; (YPiCHS 2009)	NSW; Custodial	Young people (87% male) in custody in NSW between August and October 2009; *n* = 151 ^§^	2009; 13–21 (17)	X				
Esler 2016 [70]	Qld.; Community	Indigenous young people aged 15–24 years from 11 remote north Queensland communities attending their first Young Persons Check between March 2009 and April 2011; *n* = 1883	2009–2011; 15–24 (18.8)		X			
Singh 2003 [71], Singh 2004 [72]	NT; Community	Participants of a community-wide health screening program conducted between 1992 and 1998 in an Aboriginal community on a remote island off the northern coast of Australia; *n* = 1473 (Singh 2003 *n* = 311 ^§^, Singh 2004 *n* = 210 ^§^)	1992–1998; Singh 2003: 7–17 (13.3) ^§^, Singh 2004: 4–14 (9.5) ^§^		X			
Two articles with overlapping samples:								
Schutte 2005 [36]	Central Australia, Torres Strait, North Qld.	Aboriginal people over 15 years of age from Central Australia and Torres Strait Islander people from Torres Strait and Far North Queensland communities who participated in community-based diabetes and coronary risk factor assessments between 1993 and 1995; *n* = 485 ^§^	1993–1995; 15–29 ^§^		X			
Daniel 2002 [35]	Central, northern, north-western Australia; Community	Residents over 15 years of age from 15 remote Aboriginal settlements who participated in community-based diabetes and coronary risk factor assessments between 1983 and 1997; *n* ~1450 ^§^	1989–1994; 15–34 ^§^			X		
Smith 1992 [73]	WA; Population database	Random selection of Aboriginal people aged 15–70 years in the Kimberley region of WA, identified from the WA Health Department Community Healthy Client Register as at January 1987; *n* = 118 ^§^	1988–1989; 15–29 ^§^		X		X	
**Intervention** **Studies**								
Smithers 2017 [74], Smithers 2021 [75] (Baby Teeth Talk trial)	SA; Clinic/community	Children of women who were SA residents and were either pregnant with or had given birth to an Aboriginal baby within the previous 6 weeks, recruited from January 2011 to May 2012; *n* = 454 (448 mothers)	2011–2016; W1: 2, W2: 3	X	X			
Black 2013 [76]	NSW; Clinic	Children under 17 years from 55 participating families recruited at 3 ACCHSs in NSW (Grafton, Coffs Harbour, Bowraville) between December 2008 and September 2009, with follow-up assessments between December 2009 and September 2010; *n* = 167	2008–2010; Baseline: 2–17 (7.6)	X				
Gwynn 2014 ^‡^ [77] (MRDPP)	NSW; School	Children in school years 5, 6, 7 and 8 from all primary and high schools in the Kempsey and Greater Taree regions of NSW during Summer 2007/08 (control group) and 2011/12 (intervention group); *n* = 251 control ^§^, 240 intervention ^§^	Summer 2007–2008 and 2011–2012; Years 5–8 (~10–14)	X				
**Qualitative Studies**								
Angelino 2017 [78]	Qld.; Clinic	Mother (≥18 years) or grandmother of an eligible Indigenous child (5–14 years and attended Townsville Aboriginal and Islander Health Service from 31 June 2013 until 1 July 2014), an active client of the health service, and had expressed concern with their child’s weight; *n* = 9	2013–2014; 5–14	X				

^1^: † the sample in this article combines ABC participants (80% of sample) and a non-Indigenous birth cohort; ‡ grey literature; ABC = Aboriginal Birth Cohort; LSIC = Footprints in Time: The Longitudinal Study of Indigenous Children; MRDPP = Many Rivers Diabetes Prevention Project; YPiCHS = NSW Young People in Custody Health Survey; SEARCH = Study of Environment on Aboriginal Resilience and Child Health. ^2^: NSW = New South Wales; NT = Northern Territory; Qld. = Queensland; SA = South Australia; WA = Western Australia. ^3^: § sample size of included subgroup (disaggregated by age or Indigenous status); ACCHS = Aboriginal Community Controlled Health Service; AMS = Aboriginal Medical Service; ^4^: § age of included subgroup; W1 = follow-up wave 1; ^ retrospective exposure assessment. ^5^: Outcomes: Obesity = obesity measures; BP = blood pressure; Glucose = blood glucose, insulin, or diabetes; Lipids = blood lipids; MetS = the metabolic syndrome, or cardio-metabolic risk marker clustering; ‘X’ denotes outcomes measured by study.

**Table 2:** Summary of exposures associated with cardio-metabolic risk markers among Indigenous children and adolescents, with the number of studies and direction of the association indicated.

Exposure Level	Exposures ^1^	Outcomes ^2^					Age ^3^
		Obesity	BP	Glucose	Lipids	MetS	
Individual	Higher age					~2	C,Y
	Female sex	~3	~3	~3	Ø2	~3	P,C,Y
	Higher obesity measures		↑3	↑5	↑4	↑1	P,C,Y
	Larger birth size	~4 (↑3)	Ø2	~1	~1		P,C,Y
	Smaller kidney size		↑1				C
	Maternal obesity	~2	↑1		↑1		P,C,Y
	Maternal smoking in pregnancy	~2	Ø2				P,C,Y
	Lower maternal parity	↑1					C,Y
	Higher maternal age	↑1					P,C
	Lower physical activity	↑1					C,Y
	Lower sleep duration	↑1					C
	Higher high-fat food consumption	↑1					P,C
	Higher sugar-sweetened beverage consumption	↑1					P,C
	Higher dugong consumption	↑1					C,Y
Family/Peer	Higher caregiver SBP		↑1				P,C,Y
Social	Racism	~2					C,Y
	Lower maternal education	~2					P,C
	Maternal cultural-based resilience	↑1					P,C
	Longer incarceration period ^‡^	↑1					C,Y
	No car in the household					↑1^♀^	Y
Environmental	Higher area-level SES	~4 (↑3)	↑1		~1		P,C,Y
	Less remote or urban area	~3	↑1	~1	~1	~1	P,C,Y
Interventions	Oral health intervention	~1					P

Numbers indicate the number of distinct studies reporting an association; ↑ indicates higher likelihood of the outcome; ~ indicates mixed/inconclusive results; Ø indicates consistent null associations; parentheses are used to highlight two instances where most evidence indicates an association; ♀ association only found among females. ^1^: ‡ among a custodial sample; SES = socioeconomic status. ^2^: Outcomes: Obesity = elevated obesity measures; BP = elevated blood pressure; Glucose = elevated blood glucose, insulin, or diabetes; Lipids = elevated blood lipids (or lower high-density lipoprotein cholesterol); MetS = the metabolic syndrome, or cardio-metabolic risk marker clustering. ^3^: Age group: P = preschool (0–5 years); C = childhood (6–14 years); Y = youth (15–24 years).

**Table 3:** Quantitative associations with the metabolic syndrome (MetS) or cardio-metabolic risk marker clustering, arranged by exposure type and listed in order of lowest to highest risk of bias.

Exposure	Article (Study Wave) ^1^	Main Findings (Quantitative Measure [95% CI]) ^2^	Bias ^3^
**Individual Characteristics**			
Age	Sellers 2008 (ABC W2)	No difference in mean age between those with and without MetS	H
	Campbell 2019	20–25 years (vs. 15–19 years) associated with ↑ MetS (19.8 vs. 9.7%, *p* < 0.01)	H
Sex	Sjöholm 2018 (ABC W4)	Female (vs. male) associated with ↓ ideal cardiovascular health score (3.6 vs. 4.7, *p* < 0.0001)	H
	Valery 2009	Female (vs. male) associated with ↑ MetS (15/18, or 83%, with MetS were female)	H
	Campbell 2019	Male (vs. female) associated with ↑ MetS (20.6 vs. 10.0%, *p* = 0.03)	H
Obesity measures	Sevoyan 2019 (ABC W4) ^	↑ BMI category associated with ↑ number of abnormal cardio-metabolic markers (*p* < 0.001 trend)↑ BMI (1 kg/m^2^) associated with ↑ odds of adverse cardio-metabolic profile (males aOR 1.34 [1.22, 1.47], females (aOR 1.55 [1.39, 1.73])	M
	Sellers 2008 (ABC W2)	MetS (vs. no MetS) associated with ↑ zBMI (0.67 vs. −0.89), zWC (2.69 vs. 0.27), percent body fat (30.2 vs. 19.7%), mid-arm circumferences (25.0 vs. 21.1 cm), triceps skin fold (17.6 vs. 9.5 mm), subscapular skin fold (23.2 vs. 10.0 mm), and triceps/subscapular skinfold ratio (1.3 vs. 1.0) (all *p* <0.001)	H
**Social Determinants**			
Individual SES	Sevoyan 2019 (ABC W4) ^	Among females only, car ownership (vs. no car in the household) associated with ↓ odds of adverse cardio-metabolic profile (aOR 0.28 [0.09, 0.85]) No association with years of schooling or main source of household income	M
**Environmental Factors**			
Remoteness	Sevoyan 2019 (ABC W4) *	Among females only, remote (vs. urban) associated with ↑ adverse cardio-metabolic profile (aOR 10.1 [2.76, 37.0])	M
	Sellers 2008 (ABC W2)	No association between remoteness and MetS	H

^1^: ^ non-disaggregated data (majority Indigenous); * data disaggregated for Indigenous participants within larger sample; ABC = Aboriginal Birth Cohort; W2 = follow-up wave 2. ^2^: ↑ = higher; ↓ = lower; aOR = adjusted odds ratio; BMI = body mass index; MetS = the metabolic syndrome; zBMI = BMI z-score; zWC = waist circumference z-score. ^3^: Risk of bias: H = high; M = moderate; L = low.

**Table 4:** Quantitative associations with obesity outcomes, arranged by exposure type and listed in order of lowest to highest risk of bias.

Exposure	Article (Study Wave) ^1^	Main Findings (Quantitative Measure [95% CI]) ^2^	Bias ^3^
**Individual Characteristics**			
Sex	Westrupp 2019 (LSIC W1-4)	Female (vs. male) associated with ↓ zBMI (β −0.17 [−0.28, −0.05])	L
	Thurber 2015 (LSIC W4)	No association between sex and zBMI	L
	Thurber 2017 (LSIC W4-6)	Female (vs. male) associated with ↑ rate of BMI increase (MD 0.15 kg/m^2^/year [0.07, 0.23])	L
	Sjöholm 2018 (ABC W4)	No association between sex and ideal BMI	M
	Denney-Wilson 2020	Female (vs. male) associated with ↑ odds of overweight/obese (OR 2.4)	H
	Thurber 2013 (LSIC W3-4)	No association between sex and zBMI	H
	Sjöholm 2020 (ABC W2-4)	At W3 and W4, female (vs. male) associated with ↑ elevated WHtR (W3 *p* = 0.007, W4 *p* < 0.0001)	H
Birth size	Thurber 2015 (LSIC W4)	↑ birth weight z-score (1 unit) associated with ↑ zBMI (β 0.22 [0.13, 0.31])	L
	Westrupp 2019 (LSIC W1-4)	Perinatal risk ^4^ (vs. full term, normal birth weight and not SGA) associated with ↓ zBMI (mild β −0.21 [−0.34, −0.07), moderate-to-high β −0.42 [−0.63, −0.21])	L
	Sjöholm 2021 (ABC W2-4)	Across W2-4, ↑ birth weight category (SGA, AGA, LGA) associated with ↑ BMI (*p* < 0.0001 trend), WHtR (*p* = 0.004 trend)	M
	Sayers 2007 (ABC W2)	FGR (vs. non-FGR) associated with ↓ overweight/obese (3.3 vs. 12.9%), BMI (15.7 vs. 17.3 kg/m^2^), WC (61.6 vs. 65.3 cm), mid-arm circumference (20.1 vs. 21.7 cm), triceps skin fold (8.6 vs. 11.1 mm), subscapular skin fold (28.2 vs. 37.7 mm) (all *p* < 0.01)	M
	Sayers 2011 (ABC W3)	FGR (vs. non-FGR) associated with ↓ overweight/obese (8.64 vs. 22.31%, *p* = 0.006), elevated body fat (6.17 vs. 17.69%, *p* = 0.012), BMI (19.63 vs. 22.02 kg/m^2^, *p* = 0.0006), WC (74.86 vs. 80.78 cm, *p* = 0.0009), WHtR (0.45 vs. 0.48, *p* = 0.013), percent body fat (17.43 vs. 21.60%, *p* = 0.0043), mid-arm circumference (24.94 vs. 27.25 cm, *p* = 0.0001)	H
	Sjöholm 2020 (ABC W2-4)	Across W2-4, ↑ birth weight associated with ↑ overweight/obese (W2 *p* = 0.06, W3 *p* = 0.01, W4 *p* = 0.001)	H
	Pringle 2019	↑ birth weight centile associated with ↑ BMI (β 0.02 [0.006, 0.035], R^2^ 0.12), WC (β 0.04 [0.002, 0.076], R^2^ 0.10) SGA (vs. LGA) associated with ↓ BMI (16.53 vs. 18.56 kg/m^2^, *p* = 0.052), WC (47.00 vs. 53.96 cm, *p* = 0.008)	H
	Denney-Wilson 2020	No association between birth weight and overweight/obese	H
Maternal obesity	Thurber 2015 (LSIC W4)	‘Too much’ weight gain (vs. not) associated with ↑ zBMI (β 0.18 [−0.12, 0.48])—not statistically significant	L
	Sjöholm 2018 (ABC W4)	Underweight mother (vs. normal) associated with ↑ odds of ideal BMI (aOR 2.93 [1.19, 7.21])Not statistically significant after excluding underweight participants (aOR 1.07 [0.51, 2.03])	M
	Sjöholm 2020 (ABC W2-4)	Across W2-4, ↑ maternal BMI associated with ↑ overweight/obese (W2 *p* < 0.0001, W3 *p* < 0.0001, W4 *p* = 0.004)	H
	Pringle 2019	No association between maternal body fat and BMI, WC	H
Maternal smoking	Thurber 2015 (LSIC W4)	Maternal smoking during pregnancy (vs. no smoking) associated with ↑ zBMI (β 0.25 [0.05, 0.45])	L
	Westrupp 2019 (LSIC W1-4)	No association between maternal smoking in pregnancy and zBMI	L
	Denney-Wilson 2020	No association between maternal smoking in pregnancy and overweight/obese	H
Maternal parity	Juonala 2019 (ABC W2-4)	Across W2-4, maternal parity ≥4 (vs. <4) associated with ↓ BMI (*p* = 0.039 trend)	M
	Sjöholm 2018 (ABC W4)	Maternal parity ≥6 (vs. 1) associated with ↑ odds of ideal BMI (aOR 3.75 [1.10, 12.80)Not statistically significant after excluding underweight participants (aOR 1.81 [0.70, 4.72])	M
Maternal age	Westrupp 2019 (LSIC W1-4)	↑ maternal age group associated with ↑ zBMI (β 0.51 [0.38, 0.64])	L
Diet	Thurber 2017 (LSIC W4-6)	Low consumer of high-fat food (<2 occasions on previous day vs. 2+) associated with ↓ BMI increase per year (MD −0.08 kg/m^2^/year [−0.17, 0.00]) Low sugar-sweetened beverages (vs. high, when including fruit juice) associated with ↓ BMI increase per year (MD −0.08 kg/m^2^/year [−0.16, 0.00]; when fruit juice excluded, MD −0.05 kg/m^2^/year [−0.14, 0.03])	L
	Valery 2012	Dugong consumption ≥2 times per week (vs. <2) associated with ↑ odds of overweight/obese (aOR 1.89 [1.07, 3.34]) No association with consumption of vegetables, fruit, takeaway food, fish, or turtle	M
Sleep	Fatima 2020 (LSIC W8)	“Consistently late sleepers” (vs. “early sleepers”) at W5 associated with ↑ BMI increase over follow-up (β 1.03 kg/m^2^ [0.001, 2.05])	M
	Deacon-Crouch 2018 (LSIC W7)	Sleep duration (h/weeknight) negatively correlated with age-standardized BMI (r = −0.124, *p* < 0.001)	H
Physical activity	Valery 2012	0–3 days physical activity in the last week (vs. 4–7 days) associated with ↑ odds of overweight/obese (aOR 2.50 [1.44, 4.34]), elevated WC (aOR 2.9 [1.31, 6.43])	M
**Social Determinants**			
Racism	Shepherd 2017 (LSIC W6)	Carer-perceived racism (vs. non-exposure) associated with ↑ odds of obesity (aOR 1.63 [0.98, 2.70], PAR 8.2% [2.2, 14.1])	M
	Cave 2019a and 2019b (LSIC W8)	Carer-perceived racism (vs. non-exposure) associated with ↑ odds of obesity (aOR 1.7 [1.1, 2.5])	M
	Priest 2011 (ABC W3)	No association between self-reported racism exposure and WHpR or zBMI	H
Family SES	Westrupp 2019 (LSIC W1-4)	Maternal education ≥Year 12 (vs. <12) associated with ↓ zBMI (β −0.13 [−0.24, −0.01]) No association with mother’s employment	L
	Denney-Wilson 2020	No association between maternal education ≥Year 10 (vs. <10) and overweight/obese	H
Culture	Westrupp 2019 (LSIC W1-4)	↑ maternal cultural-based resilience score associated with ↑ zBMI (β 0.12 [0.01, 0.24])	L
Incarceration	Haysom 2013 *	Incarcerated for >12 months (vs. less time) associated with ↑ odds of overweight/obese (aOR 6.92 [1.66, 28.84])	M
**Environmental Factors**			
Area-level SES	Thurber 2015 (LSIC W4)	Most disadvantaged area (vs. mid-advantaged) at W4 associated with ↓ zBMI (β −0.61 [−0.97, −0.26])	L
	Thurber 2017 (LSIC W4-6)	Most disadvantaged area (vs. most advantaged) at W3 associated with ↓ BMI (BMI intercept MD −0.52 kg/m^2^ [−0.91, −0.13])	L
	Cave 2019a (LSIC W8)	Most disadvantaged area (vs. most advantaged) at W1 associated with ↓ odds of obesity (aOR 0.2 [0.1, 0.9])	M
	Juonala 2019 (ABC W2-4)	Across W2-4, ↑ area-level disadvantage at birth associated with ↓ BMI (*p* < 0.001 trend)	M
	Sjöholm 2018 (ABC W4)	↓ area-level disadvantage (vs. highest) at birth associated with ↑ odds of ideal BMI (high disadvantage aOR 0.48 [0.25, 0.90], mid-high disadvantage aOR 0.18 [0.03, 0.44]), least disadvantage aOR 0.09 [0.02, 0.54])	M
	Sjöholm 2020 (ABC W2-4)	Across W2-4, ↑ area-level disadvantage at birth associated with ↓ overweight/obese (W2 *p* < 0.001, W3 *p* < 0.001, W4 *p* < 0.001)	H
	Spurrier 2012 *	↑ area-level advantage associated with ↑ BMI category (*p* = 0.04 trend)	H
	Denney-Wilson 2020	No association between area-level SES and overweight/obese	H
Remoteness	Westrupp 2019 (LSIC W1-4)	Non-remote (vs. remote) at W1 associated with ↓ zBMI (β −0.02 [−0.02, −0.01])	L
	Thurber 2017 (LSIC W4-6)	No association between remoteness at W3 and BMI	L
	Mackerras 2003 (ABC W2)	Urban (vs. remote) associated with ↑ BMI (17.9 vs. 15.3 kg/m^2^, *p* < 0.001), WC (66.4 vs. 60.5 cm, *p* < 0.001), mid-upper arm circumference (23.7 vs. 20.6 cm, *p* < 0.001), subscapular skinfold (10.5 vs. 7.9 mm, *p* = 0.02), triceps skinfold (11.4 vs. 8.2 mm, *p* < 0.001), and ↓ subscapular/triceps skinfolds ratio (1.0 vs. 1.1, *p* < 0.001)	M
	Juonala 2019 (ABC W2-4)	Across W2-4, urban (vs. remote) at birth associated with ↑ BMI (*p* < 0.001 trend)	M
	Sjöholm 2020 (ABC W2-4)	Across W2-4, urban (vs. remote) at birth associated with ↑ overweight/obese (W2 *p* = 0.0007, W3 *p* = 0.002, W4 *p* = 0.006)	H
	Thurber 2013 (LSIC W3-4)	At W3 and W4, urban (vs. remote) associated with ↑ zBMI (*p* < 0.001 trend)	H
	Deacon-Crouch 2018 (LSIC W7)	Remoteness negatively correlated with age-standardized BMI (r = −0.09, *p* = 0.001)	H
	Spurrier 2012 *	No association between remoteness and BMI category	H
**Interventions**			
Oral health	Smithers 2021 (BTT W2)	Immediate intervention (0–18 months vs. delayed intervention [24–36 months]) associated with ↑ zBMI (aMD 0.2 [0.0, 0.4]), mid-upper arm circumference z-score (aMD 0.2 [0.1, 0.5])	L
	Smithers 2017 (BTT W1)	No difference in obesity measures for the intervention vs. control group	M
Diet	Black 2013	No difference in BMI for the intervention vs. control group	H
Behaviors	Gwynn 2014	No difference in BMI for the intervention vs. control group	H

^1^: * data disaggregated for Indigenous participants within larger sample; ABC = Aboriginal Birth Cohort; BTT = Baby Teeth Talk trial; LSIC = Longitudinal Study of Indigenous Children; W1 = follow-up wave 1. ^2^: ↑ = higher; ↓ = lower; β = linear regression coefficient; AGA = appropriate for gestational age; aMD = adjusted mean difference; aOR = adjusted odds ratio; BMI = body mass index; FGR = fetal growth restriction; LGA = large for gestational age; MD = mean difference; OR = odds ratio; PAR = population attributable risk; r = correlation coefficient; R^2^ = coefficient of determination (proportion of total variation in the outcome measure accounted for by the exposure); SES = socioeconomic status; SGA = small for gestational age; WC = waist circumference; WHtR = waist-to-height ratio; WHpR = waist-to-hip ratio; zBMI = BMI z-score; zWC = WC z-score. ^3^: Risk of bias: H = high; M = moderate; L = low. ^4^: Perinatal risk: ‘moderate-to-high’ if born very pre-term (<32 weeks), and/or extremely small for gestational age (<2nd percentile), and/or very low birthweight (<1500 g) [classification based on category that placed the child at greatest risk, regardless of whether this was based on one or all three measures of perinatal risk]; ‘mild’ if born at 32–36 weeks, and/or small for gestational age (2nd–9th percentile), and/or had birthweight 1500–2499g; vs. full-term (≥37 week’s gestation), normal birthweight (≥2500 g), and not small for gestational age (≥10th percentile).

**Table 5:** Quantitative associations with blood pressure outcomes, arranged by exposure type and listed in order of lowest to highest risk of bias.

Exposure	Article (Study Wave) ^1^	Main Findings (Quantitative Measure [95% CI]) ^2^	Bias ^3^
**Individual Characteristics**			
Sex	Mann 2015 (ABC W3)	Female (vs. male) associated with ↓ SBP (−5.40 mmHg [−7.48, −3.06]; β* −0.23)	M
	Sjöholm 2018 (ABC W4)	Female (vs. male) associated with ↑ odds of ideal BP (aOR 5.51 [2.84, 10.7])	M
	Larkins 2017 (SEARCH base)	No association between sex and blood pressure	M
	Esler 2016	Male (vs. female) associated with ↑ odds of HT (aOR 4.37 [2.92, 6.54])	H
Obesity measures	Gialamas 2018 (ABC W2-3)	↑ zBMI at W2 associated with ↑ SBP at W2 (males β 1.89 mmHg [1.05, 2.73], females β 1.74 [0.76, 2.73]), SBP at W3 (males β 1.43 [0.54, 2.33], females β 1.09 [0.06, 2.12]), DBP (males only) at W2 and W3 (β 0.71 for both)↑ zBMI at W3 associated with ↑ SBP at W3 (males β 1.53 [0.59, 2.48], females β 1.49 [0.43, 2.55]), DBP at W3 (males β 0.85 [0.23, 1.48], females β 0.98 [0.24,1.65])	L
	Larkins 2017 (SEARCH base)	↑ zBMI associated with ↑ zDBP (β 0.08 [0.01, 0.15]), zSBP (β 0.08 [−0.01, 0.16])	M
	Mann 2015 (ABC W3)	↑ BMI (1 kg/m^2^) at W3 associated with ↑ SBP (0.61 mmHg [0.27, 0.96]; β* 0.32), DBP (0.47 [0.23, 0.71])	M
	Sayers 2009 (ABC W2)	↑ weight (1 kg) at W2 associated with ↑ SBP ^§^ (β 0.0042 [0.0030, 0.0054]), DBP (β 0.20 [0.11, 0.30])	M
	Sevoyan 2019 (ABC W4) ^	↑ BMI category associated with ↑ elevated BP (*p* <0.001 trend)	H
	Esler 2016	Overweight, obese (vs. normal) associated with ↑ odds of HT (aOR 2.46 [1.53, 3.97]; aOR 4.59 [2.87, 7.36])	H
Birth size	Gialamas 2018 (ABC W2-3)	No association between blood pressure and birth weight or length	L
	Mann 2015 (ABC W3)	Indirect effect of birth weight on SBP (β* 0.09) mediated through BMI at W3	M
	Sayers 2009 (ABC W2)	↑ birth weight (1 kg) associated with ↑ SBP ^§^ (β −0.030 [−0.046, −0.013]), DBP (β −1.70 [−3.01, −0.38])	M
	Sjöholm 2021 (ABC W2-4)	At W4 only, ↑ birth weight category (SGA, AGA, LGA) associated with ↑ SBP (109.0, 112.0, 113.7 mmHg), DBP (69.9, 71.9 mmHg [SGA, AGA only])Associations did not persist after adjusting for current BMI, indicating potential mediation	M
	Sjöholm 2018 (ABC W4)	No association between blood pressure and birth weight	M
	Singh 2003 *	No association between blood pressure and birth weight, before or after taking current weight into account	M
Kidney size	Singh 2004 *	↑ kidney length (1 cm) associated with ↓ SBP (−3.2 mmHg) ↑ kidney volume (10 mL) associated with ↓ SBP (−1.1 mmHg)	M
Maternal obesity	Sjöholm 2018 (ABC W4)	Obese mother (vs. normal) associated with ↓ odds of ideal BP (aOR 0.13 [0.03, 0.62])	M
Maternal smoking	Mann 2015 (ABC W3)	No association between maternal smoking during pregnancy and blood pressure	M
	Larkins 2017 (SEARCH base)	No association between maternal smoking during pregnancy and blood pressure	M
**Family/Peer Factors**			
Caregiver SBP	Larkins 2017 (SEARCH base)	↑ caregiver SBP (per 10 mmHg) associated with ↑ child zSBP (β 0.15 [0.07, 0.24]), zDBP (β 0.08 [0.01, 0.15])	M
**Environmental Factors**			
Area-level SES	Juonala 2019 (ABC W2-4)	Across W3-4, ↑ area-level disadvantage at birth associated with ↓ SBP (*p* = 0.022 trend)	M
	Sjöholm 2018 (ABC W4)	↓ area-level disadvantage category (vs. highest) at birth associated with ↓ odds of ideal BP (high disadvantage aOR 0.38 [0.18, 0.79]; mid-high disadvantage aOR 0.12 [0.03, 0.49]; least disadvantage aOR 0.05 [0.01, 0.32])	M
Remoteness	Mackerras 2003 (ABC W2)	Urban (vs. remote) associated with ↑ SBP (109.6 vs. 106.2 mmHg, *p* = 0.004)	M
	Mann 2015 (ABC W3)	Remote (vs. urban) at W3 associated with ↓ SBP (−3.16 mmHg [−6.14, −0.018]; β* 0.14)	M
	Sjöholm 2018 (ABC W4)	Urban (vs. remote) associated with ↓ odds of ideal BP (aOR 0.11 [0.02, 0.76])	M
**Interventions**			
Oral health intervention	Smithers 2021 (BTT W2)	No association between blood pressure and oral health intervention group	L
	Smithers 2017 (BTT W1)	No association between blood pressure and oral health intervention group	M

^1^: ^ non-disaggregated data (majority Indigenous); * data disaggregated for participants aged <25 years within larger sample; ABC = Aboriginal Birth Cohort; BTT = Baby Teeth Talk trial; SEARCH = Study of Environment on Aboriginal Resilience and Child Health; W2 = follow-up wave 2. ^2^: ↑ = higher; ↓ = lower; β = linear regression coefficient; β* = standardized regression coefficient from pathway analysis; § = outcome measure log-transformed; AGA = appropriate for gestational age; aOR = adjusted odds ratio; BMI = body mass index; BP = blood pressure; DBP = diastolic BP; HT = hypertension; LGA = large for gestational age; SBP = systolic BP; SGA = small for gestational age; zBMI = BMI z-score; zDBP = DBP z-score; zSBP = SBP z-score. ^3^: Risk of bias: H = high; M = moderate; L = low.

**Table 6:** Quantitative associations with glucose, insulin and diabetes outcomes, arranged by exposure type and listed in order of lowest to highest risk of bias.

Exposure	Article (Study Wave) ^1^	Main Findings (Quantitative Measure [95% CI]) ^2^	Bias ^3^
**Individual Characteristics**			
Sex	Sjöholm 2018 (ABC W4)	No association between sex and HbA1c	M
	Riley 2021 (SEARCH W2)	No association between sex and HbA1c	M
	Braun 1996	Female (vs. male) associated with ↑ fasting and 2 h insulin (*p* < 0.05 trend)	H
Obesity measures	Sayers 2004 (ABC W2)	↑ weight (1 kg) and height (1 cm) at W2 associated with ↑ fasting insulin ^§^ (ratio 1.02 [1.01, 1.02]), HOMA-IR (1.02 [1.01, 1.02]), fasting glucose ^§^ (1.001 [1.001, 1.002])	M
	Sayers 2009 (ABC W2)	↑ weight (1 kg) at W2 associated with ↑ fasting insulin ^§^ (β 0.037 [0.028, 0.046]), fasting glucose (β 0.011 [0.0036, 0.019])	M
	Sayers 2013 (ABC W3)	↑ weight (1 kg) at W3 associated with ↑ fasting insulin ^§^ (ratio 1.03 [1.02, 1.03]; R^2^ 0.299), HOMA-IR ^§^ (1.03 [1.02, 1.04]), fasting glucose ^§^ (1.001 [1.001, 1.003]; R^2^ 0.070)↑ height (1 cm) at W3 associated with ↑ fasting insulin ^§^ (ratio 1.03 [1.01, 1.05]; R^2^ 0.055), HOMA-IR ^§^ (1.03 [1.01, 1.06]) ↑ BMI (1 kg/m^2^) at W3 associated with ↑ fasting insulin ^§^ (ratio 1.09 [1.07, 1.12]), HOMA-IR ^§^ (1.10 [1.08, 1.13]), fasting glucose ^§^ (1.007 [1.003, 1.01])	M
	Sellers 2008 (ABC W2)	zWC, zBMI positively correlated with HOMA-IR (r = 0.37, r = 0.29; *p* < 0.001)	H
	Sevoyan 2019 (ABC W4) ^	↑ BMI category associated with ↑ elevated HbA1c (*p* < 0.001 trend)	H
	Riley 2021 (SEARCH W2)	Obesity (vs. normal) associated with ↑ elevated HbA1c (aPR 2.52 [0.73, 8.63])—not statistically significant	H
	Valery 2009	BMI, WC positively correlated with HOMA-IR (r = 0.54, r = 0.72; *p* < 0.001) Overweight/obese (vs. normal) associated with ↑ HOMA-IR (3.58 vs. 2.25, *p* = 0.002), elevated fasting insulin (56 vs. 30%, *p* = 0.021), mean fasting insulin (18.74 vs. 11.96 mU/L, *p* = 0.001), mean HbA1c (5.55 vs. 5.39%, *p* = 0.037)	H
	Daniel 2002 *	↑ BMI category (22–24.9, 25–29.9, 30–34.9, ≥35 vs. <22 kg/m^2^) associated with ↑ odds of IGT (males: OR 3.3 [1.2, 9.9], 7.3 [2.9, 20.4], 11.4 [3.6, 36.6], 12.5 [3.2, 45.6]; females: OR 4.0 [1.5, 11.9], 6.1 [2.5, 16.6], 5.3 [1.7, 16.8], 9.3 [3.1, 29.0]), diabetes (males: OR 1.9 [0.3, 11.1], 6.2 [1.7, 28.6], 9.4 [1.9, 51.6], 8.1 [0.9, 56.3]; females: OR 10.3 [2.5, 69.5), 10.1 [2.6, 65.8], 25.7 [6.4, 168.1], 21.2 [4.7, 147.5])	H
	Braun 1996	↑ BMI at baseline associated with fasting insulin in upper tertile (vs. lower) at baseline (*p* < 0.05 trend), 2 h insulin in upper tertile at baseline ↑ BMI at follow-up associated with 2 h insulin in upper tertile (vs. lower) at follow-up (24.2 vs. 19.5 kg/m^2^, *p* < 0.05), abnormal glucose tolerance (IGT or T2DM vs. normal tolerance) at follow-up (25.6 vs. 20.8 kg/m^2^, *p* < 0.05)	H
Birth size	Sayers 2004 (ABC W2)	↑ birth weight (500 g) associated with ↑ fasting insulin ^§^ (ratio 1.04 [1.0, 1.1]), before adjusting for current child sizeAssociations did not persist after adjusting for current height or weight, indicating potential mediation	M
	Sayers 2009 (ABC W2)	No association between birth weight and insulin or glucose levels, before or after adjusting for current weight	M
	Sayers 2013 (ABC W3)	↑ birth weight (1 kg) associated with ↑ fasting glucose ^§^ (ratio 1.07 [1.03, 1.11]; R^2^ 0.07) FGR (vs. non-FGR) associated with ↓ fasting glucose ^§^ (ratio 0.93 [0.89, 0.98]; R^2^ 0.06) Positive and significant interactions between birth weight and height for insulin (*p* = 0.006) and HOMA-IR (*p* = 0.015)	M
	Sjöholm 2018 (ABC W4)	No association between birth weight and ideal HbA1c	M
**Environmental Factors**			
Remoteness	Mackerras 2003 (ABC W2)	Urban (vs. remote) associated with ↑ fasting insulin (7 vs. 4 mU/L, *p* = 0.007) No association between remoteness and fasting glucose	M
	Sjöholm 2018 (ABC W4)	No association between mother’s remoteness at birth and ideal HbA1c	M

^1^: ^ non-disaggregated data (majority Indigenous); * non-disaggregated data (majority aged <25 years); ABC = Aboriginal Birth Cohort; SEARCH = Study of Environment on Aboriginal Resilience and Child Health; W2 = follow-up wave 2. ^2^: ↑ = higher; ↓ = lower; β = linear regression coefficient; § = outcome measure log-transformed; aPR = adjusted prevalence ratio; BMI = body mass index; FGR = fetal growth restriction; HbA1c = glycated hemoglobin; HOMA-IR = Homeostasis Model Assessment of Insulin Resistance score; IGT = impaired glucose tolerance; OR = odds ratio; r = correlation coefficient; R^2^ = coefficient of determination (proportion of total variation in the outcome measure accounted for by the exposure); T2DM = type 2 diabetes mellitus; WC = waist circumference; zBMI = BMI z-score; zWC = WC z-score. ^3^: Risk of bias: H = high; M = moderate; L = low.

**Table 7:** Quantitative associations with lipid outcomes, arranged by exposure type and listed in order of lowest to highest risk of bias.

Exposure	Article (Study Wave) ^1^	Main Findings (Quantitative Measure [95% CI]) ^2^	Bias ^3^
**Individual Characteristics**			
Sex	Riley 2021 (SEARCH W2)	Female (vs. male) associated with ↑ low HDL-c (aPR 1.54 [0.97, 2.47])—not statistically significant	M
	Sjöholm 2018 (ABC W4)	No association between sex and ideal TotChol	M
Obesity measures	Gialamas 2018 (ABC W2-3)	Among males, ↑ zBMI at W2 associated with ↑ TotChol at W3 (β 0.12 mmol/L [0.05, 0.19]), LDL-c at W3 (β 0.09 [0.03, 0.15]) ↑ zBMI at W3 associated with ↑ TotChol at W3 (males only, β 0.12 [0.05, 0.19]), ↓ HDL-c (females only, β −0.04 [−0.05, −0.02])	L
	Sayers 2009 (ABC W2)	↑ weight (1 kg) at W2 associated with ↑ TotChol ^§^ (β 0.0021 [0.00033, 0.0039]), fasting TG ^§^ (β 0.0065 [0.00046, 0.012])	M
	Sevoyan 2019 (ABC W4) ^	↑ BMI category associated with ↑ elevated TG (*p* < 0.001 trend), low HDL-c (females *p* <0.05 trend, males *p* = 0.17 trend)	H
	Riley 2021 (SEARCH W2)	Obesity (vs. normal) associated with ↑ elevated TotChol (aPR 1.28 [1.06, 1.54]), low HDL-c (aPR 2.00 [1.19, 3.35]), elevated LDL-c (aPR 1.14 [0.96, 1.35])	H
	Valery 2009	Overweight/obese (vs. normal) associated with ↑ low HDL-c (63% vs. 41%, *p* = 0.049), elevated TG (20 vs. 7%, *p* = 0.134)	H
	Smith 1992 *	↑ BMI (1 kg/m^2^) associated with ↑ TotChol (males β 0.062 ± SE 0.032 mmol/L, females β 0.053 ± SE 0.015)	H
Birth size	Sjöholm 2021 (ABC W2-4)	At W2 only, ↑ birth weight category (SGA, AGA, LGA) associated with ↑ TG (1.09, 1.20, 1.50 mmol/L) Associations did not persist after adjusting for current BMI, indicating potential mediation	M
	Sayers 2009 (ABC W2)	No association between birth weight and lipids (TotChol, HDL-c, LDL-c, TG), before or after adjusting for current weight	M
Maternal obesity	Sjöholm 2018 (ABC W4)	Obese mother (vs. normal) associated with ↓ odds of ideal TotChol (aOR 0.13 [0.03, 0.58])	M
**Environmental Factors**			
Area-level SES	Juonala 2019 (ABC W2-4)	Across W2-4, ↑ area-level disadvantage at birth associated with ↓ HDL-c (*p* < 0.001 trend) Across W3-4, ↑ area-level disadvantage at birth associated with ↓ LDL-c (*p* = 0.010 trend)	M
	Sjöholm 2018 (ABC W4)	No association between area-level SES at birth and ideal TotChol	M
Remoteness	Mackerras 2003 (ABC W2)	Urban (vs. remote) associated with ↑ TotChol (4.3 vs. 4.0 mmol/L, *p* < 0.001), HDL-c (1.4 vs. 1.2 mmol/L, P <0.001)	M
	Juonala 2019 (ABC W2-4)	Across W3-4, urban (vs. remote) at birth associated with ↑ HDL-c (*p* < 0.001 trend), ↓ TG (*p* = 0.043 trend)	M
	Sjöholm 2018 (ABC W4)	No association between remoteness at birth and ideal TotChol	M

^1^: ^ non-disaggregated data (majority Indigenous); * non-disaggregated data (majority aged <25 years); ABC = Aboriginal Birth Cohort; SEARCH = Study of Environment on Aboriginal Resilience and Child Health; W2 = follow-up wave 2. ^2^: ↑ = higher; ↓ = lower; β = linear regression coefficient; § = outcome measure log-transformed; aPR = adjusted prevalence ratio; BMI = body mass index; HDL-c = high-density lipoprotein cholesterol; LDL-c = low-density lipoprotein cholesterol; SE = standard error; TG = triglycerides; TotChol = total cholesterol; zBMI = BMI z-score. **^3^**: Risk of bias: H = high; M = moderate; L = low.

## Data Availability

Not applicable.

## Supplementary Materials

The following supporting information can be downloaded at: https://www.mdpi.com/article/10.3390/ijerph19159180/s1, Table S1: MEDLINE search strategy; Table S2: Extracted data not included in qualitative synthesis due to high risk of bias and no comparable data for the same exposures from other studies, presented by outcome; Table S3: Exposure–outcome associations investigated in individual articles where no association was found, ordered by risk of bias; Table S4: Results from the NHLBI quality assessment tool for observational cohort and cross-sectional studies; Table S5: Results from the NHLBI quality assessment tool for controlled intervention studies; Table S6: Results from the NHLBI quality assessment tool for before–after (pre-post) studies; Table S7: Aboriginal and Torres Strait Islander Quality Appraisal Tool results.
