# Supplementary material for: Potential Determinants of Cardio-Metabolic Risk among Aboriginal and Torres Strait Islander Children and Adolescents: A Systematic Review
Source: Int J Environ Res Public Health. 2022 Jul 27;19(15):9180. doi: 10.3390/ijerph19159180 (PMC9368168; doi:10.3390/ijerph19159180)
Supplement: Supplementary file 1 [file ijerph-19-09180-s001.zip › ijerph-1800779-supplementary.pdf]

Table S1. MEDLINE search strategy.

1. metabolic syndrome/ OR exp diabetes mellitus/ OR diabetes mellitus, type 2/ OR prediabetic state/ OR hyperglycemia/ OR glucose intolerance/ OR hyperinsulinism/ OR insulin resistance/ OR lipid metabolism disorders/ OR dyslipidemias/ OR hyperlipidemias/ OR cardiovascular diseases/ OR vascular diseases/ OR atherosclerosis/ OR exp hypertension/ OR blood pressure/ OR obesity/ OR body weight/ OR overweight/ OR pediatric obesity/ OR adipose tissue/ OR abdominal fat/ OR body fat distribution/ OR adiposity/ OR body mass index/ OR waist circumference/ OR waist-hip ratio/
2. metabolic syndrome OR metabolic dysfunction OR metabolic risk* OR metabolic abnormal* OR cardiometabolic disease* OR cardiometabolic disorder* OR cardiometabolic risk* OR cardiovascular disease* OR cardiovascular disorder* OR cardiovascular risk* OR vascular disease* OR vascular disorder* OR vascular risk* OR syndrome x OR visceral fat syndrome OR deadly quartet OR risk factor clustering OR obese OR obesity OR overweight OR central fat OR abdominal fat OR visceral fat OR body fat OR central adipos* OR abdominal adipos* OR visceral adipos* OR waist OR abdominal circumference OR body mass OR BMI OR blood pressure OR hypertensi* OR cholesterol OR triglycerid* OR high density lipoprotein OR low density lipoprotein OR ((blood OR abnormal OR lower*) ADJ2 lipid*) OR dyslipid?emi* OR hyperlipid* OR hypertriglycerid* OR hypercholesterol* OR diabet* OR prediabet* OR blood glucose OR plasma glucose OR impaired glucose OR glucose intoleran* OR glucose toleran* OR (fasting ADJ2 glucose) OR glycated h?emoglobin OR hba1c OR impaired fasting gl* OR glyc?emia OR hyperglyc?emi* OR dysglyc?emi* OR OGTT OR hyperinsulin* OR insulin insens* OR insulin resistan* OR insulin sensitiv*
3. 1 OR 2
4. Oceanic Ancestry Group/
5. indigen* OR torres strait* OR aborigin* OR first nation*
6. 4 OR 5
7. exp Australia/
8. australia* OR new south wales OR NSW OR queensland OR victoria* OR tasmania* OR northern territory OR top end
9. 7 OR 8
10. 6 AND 9
11. Adolescent/ OR Young Adult/ OR Child/ OR Infant/ OR Minors/ OR Students/
12. child* OR girls OR girl OR boys OR boy OR adolesc* OR teen OR teens OR teenage* OR youth* OR early adult* OR emerging adult* OR young adult* OR (young ADJ2 (people or person*)) OR juvenile* OR p?ediat* OR toddler* OR infant* OR youngster* OR kid OR kids OR preadolescen* OR puberty OR prepuberty OR pubescen* OR prepubescen* OR underage* OR under age* OR student* OR school age* OR schoolage* OR school child* OR schoolchild* OR schoolgirl* OR schoolboy*
13. 11 OR 12
14. 3 AND 10 AND 13

Table S2. Extracted data not included in qualitative synthesis due to high risk of bias and no comparable data for the same exposures from other studies, presented by outcome.

Outcome	Article (Study wave) [1]	Exposure and main findings [2]	Bias [3]
MetS	Juonala 2016 (ABC W3)	**Previous MetS:** MetS (vs no MetS) at W2 associated with ↑ MetS at W3 (35.4 vs 13.6%, P <0.001)	H
	Valery 2009	**MetS components:** MetS (vs no MetS) associated with ↑ low HDL-c, elevated insulin (17/18 [94%] had each of low HDL-c and elevated insulin)	H
Obesity	Sellers 2008 (ABC W2)	**BMI/WC:** 100% of children classified as overweight or obese based on BMI had an elevated WC; however, 58.7% of children with an elevated WC were not classified as either overweight or obese based on BMI	H
	Juonala 2016 (ABC W3)	**Previous elevated WC:** elevated WC (vs normal) at W2 associated with ↑ elevated WC at W3 (58.9 vs 12.3%, P <0.001)	H
	Sjöholm 2020 (ABC W2-4)	**Age:** ↑ age across W2-4 associated with ↑ overweight/obese, elevated WHtR (P <0.0001 trend)	H
	Sevoyan 2019 (ABC W4)	**BMI/WC ^:** ↑ BMI category associated with ↑ elevated WC (P <0.001 trend)<br>**BMI/WC *:** Elevated WC was present in all Indigenous females in the overweight and obese categories (100%), as well as 59% and 75% of those with normal BMI in remote and urban areas, respectively.	H
	Thurber 2013 (LSIC W3-4)	**Previous BMI:** zBMI at W3 positively correlated with zBMI at W4 for older cohort (r = 0.56)	H
	Webster 2013 (Gudaga W1)	**Rapid weight gain (RWG):** RWG in the first year of life (vs no RWG) associated with ↑ overweight/obese (61.9 vs 23.8%, P <0.001)	H
	Denney-Wilson 2020 (Gudaga W2)	**RWG:** RWG in the first year of life (vs no RWG) associated with ↑ overweight/obese (OR 2.7, P <0.006)<br>**Previous BMI:** BMI >85th percentile at 2 years of age (vs below) associated with ↑ overweight/obese (OR 3.9, P <0.002)	H
Blood pressure	Juonala 2016 (ABC W3)	**Previous elevated BP:** Elevated BP (vs normal) at W2 associated with ↑ elevated BP at W3 (41.7% vs 22.3%, P <0.001)	H
	Esler 2016	**Lipids:** elevated TG (vs normal) associated with ↑ odds of HT (aOR 2.38 [95% CI 1.61, 3.52])	H
	Schutte 2005	**Insulin †:** Positive correlation between fasting insulin and mean arterial pressure (Aboriginal: $r_{partial}$ = 0.110; Torres Strait Islander: $r_{partial}$ = 0.257)	H

Outcome	Article (Study wave) [1]	Exposure and main findings [2]	Bias [3]
	Smith 1992	**Alcohol †:** Among males only, self-reported drinker (vs non-drinker) associated with ↑ SBP (+15.8 mmHg), DBP (+16 mmHg); Among males only, circulating gamma-glutamyl transpeptidase (alcohol marker) above 50 U/L (vs <50 U/L) associated with ↑ SBP (+9.9 mmHg), DBP (+9.9 mmHg)	H
Glucose, insulin, or diabetes	Juonala 2016 (ABC W3)	**Previous elevated glucose:** elevated glucose (vs normal) at W2 was not associated with elevated glucose at W3	H
	Braun 1996	**Age:** Fasting insulin was higher at follow-up (18 years) than baseline (11 years) (mean [±SE], 117 [±118] vs 75 [±49] pmol/L); Fasting glucose was higher at baseline than follow-up (4.9 [±1.1] vs 4.4 [±1.3] mmol/L); During OGTT, 2-h insulin and 2-h glucose were higher at follow-up than baseline (P <0.05 trend) **Lipids:** ↑ TG at follow-up associated with fasting insulin in upper tertile (vs lower) at follow-up (1.64 vs 1.30 mmol/L, P <0.05), abnormal glucose tolerance (vs normal tolerance) at follow-up (2.73 vs 1.16 mmol/L, P <0.05); ↑ TotChol at follow-up associated with abnormal glucose tolerance (vs normal tolerance) at follow-up (5.51 vs 4.55 mmol/L, P <0.02)	H
Lipids	Juonala 2016 (ABC W3)	**Previous low HDL-c:** low HDL-c (vs normal) at W2 associated with ↑ low HDL-c at W3 (41.1% vs 19.2%, P <0.001) **Previous elevated TG:** elevated TG (vs normal) at W2 was not associated with elevated TG at W3	H

1: ABC = Aboriginal Birth Cohort; LSIC = Longitudinal Study of Indigenous Children; W1 = follow-up wave 1. **2:** ^ non-disaggregated data (majority Indigenous); * data disaggregated for Indigenous participants within larger sample; **†** non-disaggregated data (majority aged <25 years); ↑ = higher; ↓ = lower; aOR = adjusted odds ratio; BMI = body mass index; BP = blood pressure; DBP = diastolic BP; HDL-c = high-density lipoprotein cholesterol; HT = hypertension; MetS = the metabolic syndrome; OGTT = oral glucose tolerance test; OR = odds ratio; SBP = systolic BP; TG = triglycerides; TotChol = total cholesterol; WC = waist circumference; zBMI = BMI z-score. **3:** Risk of bias: H = high; M = moderate; L = low.

Table S3. Exposure-outcome associations investigated in individual articles where no association was found, ordered by risk of bias.

Article (Study wave) [1]	Outcome [2]	Exposures	Bias [3]
Gialamas 2018 (ABC W2-3)	1. Blood pressure<br>2. Lipids (TotChol, HDL-c, LDL-c)	1. Birth weight, birth length<br>2. Birth weight, birth length, height, leg length, trunk length, leg-to-trunk ratio	L
Thurber 2015 (LSIC W4)	Obesity (zBMI)	Age, sex	L
Thurber 2017 (LSIC W4-6)	Obesity (rate of BMI change)	Screen time (W3), remoteness (W3)	L
Westrupp 2019 (LSIC W1-4)	Obesity (zBMI)	Singleton birth, maternal factors (smoking, drinking, social & emotional wellbeing, stressful life events, Indigenous status), mother employed (W1-4)	L
Sayers 2004 (ABC W2)	Glucose (glucose, insulin, HOMA-IR)	Birth weight, ponderal index, birth weight for gestational age	M
Sayers 2007 (ABC W2)	Obesity	Ponderal index	M
Sayers 2009 (ABC W2)	1. Blood pressure<br>2. Glucose (glucose, insulin)<br>3. Lipids (TotChol, HDL-c, LDL-c, TG)	1. Sex<br>2. Birth weight<br>3. Birth weight	M
Mann 2015 (ABC W3)	Blood pressure	Gestational age, maternal smoking, smoking (W3)	M
Juonala 2019 (ABC W2-4)	1. Blood pressure<br>2. Lipids (HDL-c, LDL-c, TG)	1. Maternal parity, remoteness (birth)<br>2. Maternal parity	M
Sjöholm 2018 (ABC W4)	1. Obesity (ideal BMI)<br>2. Blood pressure (ideal BP)<br>3. Glucose (ideal HbA1c)<br>4. Lipids (ideal TotChol)	1. Sex, birth weight, remoteness (birth), household size (birth)<br>2. Birth weight, maternal parity, household size (birth)<br>3. Sex, birth weight, maternal BMI, maternal parity, area-level SES (birth), remoteness (birth), household size (birth)<br>4. Sex, birth weight, maternal parity, area-level SES (birth), remoteness (birth), household size (birth)	M
Shepherd 2017 (LSIC W6)	Obesity (BMI category)	Primary carer & family racism experience (W1-5)	M

Article (Study wave) [1]	Outcome [2]	Exposures	Bias [3]
Cave 2019b (LSIC W8)	Obesity (BMI category)	Age of first racism exposure	M
Larkins 2017 (SEARCH baseline)	Blood pressure	Age, sex, WHtR, maternal smoking, breastfed, diet (serves of vegetables, fruit, high-salt snacks), physical activity, emotional difficulties, family income, caregiver education, caregiver job loss, crowded living conditions	M
Riley 2021 (SEARCH W2)	1. Glucose (elevated HbA1c)<br>2. Lipids (TotChol, HDL-c, LDL-c)	1. Age, sex<br>2. Age	M
Haysom 2013	Obesity (BMI category)	Gender, age, exercise before incarceration, exercise since incarcerated, smoking before incarceration, alcohol use before incarceration, experienced out-of-home care, psychological disorder, psychotropic medication, self-perceived overweight, self-perceived weight gain since incarcerated	M
Valery 2012	Obesity (BMI category, elevated WC)	Dietary intake (fruit, vegetables, takeaway foods, turtle, fish)	M
Singh 2003	Blood pressure	Birth weight	M
Sellers 2008 (ABC W2)	MetS	Age, birth weight, gestational age, pubertal onset, mother remoteness (birth), remoteness (W2)	H
Priest 2011 (ABC W3)	Obesity (WHpR, zBMI)	Racism experience (W3, self-reported)	H
Sjöholm 2020 (ABC W2-4)	Obesity (BMI)	Sex	H
Sevoyan 2019 (ABC W4)	MetS (adverse cardiometabolic profile)	Years of schooling, main source of household income	H
Thurber 2013 (LSIC W3-4)	Obesity (zBMI)	Sex	H
Deacon-Crouch 2018 (LSIC W7)	Obesity (BMI)	Area-level SES	H
Denney-Wilson 2020 (Gudaga W2)	Obesity (BMI category)	Maternal age, maternal smoking, low birth weight, prematurity, SGA, breastfeeding in first year of life, mother marital status, mother education, area-level SES	H
Campbell 2019	MetS, Obesity (BMI category)	Age at first exposure to antibiotics within first 24 months of life, no. of prescriptions for antibiotics in first 24 months of life	H
Pringle 2019	Obesity (BMI)	Maternal factors (percentage body fat, visceral fat, plasma glucose)	H

Article (Study wave) [1]	Outcome [2]	Exposures	Bias [3]
Spurrier 2012	Obesity (BMI category)	Sex, remoteness	H
Braun 1996	Glucose (glucose, insulin, IGT, T2DM)	Pubertal staging at follow-up, elevated insulin during an OGTT at baseline did not predict the development of IGT or T2D at follow-up	H
Smith 1992	Blood pressure	Tobacco use	H

1: ABC = Aboriginal Birth Cohort; LSIC = Longitudinal Study of Indigenous Children; SEARCH = Study of Environment on Aboriginal Resilience and Child Health; W1 = follow-up wave 1. **2:** BMI = body mass index; BP = blood pressure; HbA1c = glycated hemoglobin; HDL-c = high-density lipoprotein cholesterol; HOMA-IR = Homeostasis Model Assessment of Insulin Resistance score; IGT = impaired glucose tolerance; MetS = the metabolic syndrome; T2DM = type 2 diabetes mellitus; TG = triglycerides; TotChol = total cholesterol; WC = waist circumference; WHpR = waist-to-hip ratio; zBMI = BMI z-score. **3:** Risk of bias: H = high; M = moderate; L = low.

Table S4. Results from the NHLBI quality assessment tool for observational cohort and cross-sectional studies.

Article ID	Design	Criteria number														Risk of bias	Main bias issues
		1	2	3	4	5	6	7	8	9	10	11	12	13	14		
Gialamas 2018	L/ CS	Y	Y	Y	Y	N	Y	Y	Y	Y	Y	Y	CD	N	Y	Low	Bias acknowledged/managed
Thurber 2015	L	Y	Y	CD	Y	N	Y	Y	Y	N	NA	Y	CD	N	Y	Low	Bias acknowledged/managed
Thurber 2017	L	Y	Y	CD	Y	N	Y	Y	N	N	N	Y	CD	N	Y	Low	Bias acknowledged/managed
Westrupp 2019	L	Y	Y	CD	Y	N	Y	Y	Y	CD	NA	Y	CD	Y	N	Low	Bias acknowledged/managed
Juonala 2019	L	Y	Y	Y	Y	N	Y	Y	Y	Y	Y	Y	CD	N	Y	Moderate	Potential for selection bias from LTFU/missing data
Mann 2015	L	Y	Y	Y	Y	N	Y	Y	Y	Y	Y	Y	CD	N	Y	Moderate	Potential for selection bias from LTFU/missing data
Sayers 2004	L/ CS	Y	Y	Y	Y	N	Y	Y	Y	Y	N	Y	Y	Y	N	Moderate	Potential for residual confounding of both longitudinal birth exposures (e.g. by maternal factors, remoteness/SES) and cross-sectional exposures (+ behavioral factors)
Sayers 2007	L	Y	Y	Y	Y	N	Y	Y	N	Y	N	Y	CD	Y	Y	Moderate	Potential for residual confounding (e.g. by maternal factors)
Sayers 2009	L/ CS	Y	Y	Y	Y	N	Y	Y	Y	Y	NA	Y	CD	Y	Y	Moderate	Potential for residual confounding of both longitudinal birth exposures (e.g. by maternal factors) and cross-sectional exposures (+ behavioral factors)
Sayers 2013	L/ CS	Y	Y	Y	Y	N	Y	Y	Y	Y	NA	Y	CD	N	Y	Moderate	Potential for selection bias from LTFU/missing data; potential for residual confounding of both longitudinal birth exposures (e.g. by maternal factors) and cross-sectional exposures (+ behavioral factors)
Sjoholm 2018	L	Y	Y	Y	Y	N	Y	Y	Y	Y	N	Y	CD	N	Y	Moderate	Potential for selection bias from LTFU/missing data

Article ID	Design	Criteria number														Risk of bias	Main bias issues
		1	2	3	4	5	6	7	8	9	10	11	12	13	14		
Sjoholm 2021	L	Y	Y	Y	Y	N	Y	Y	Y	Y	N	Y	CD	N	N	Moderate	Potential for selection bias from LTFU/missing data; potential for residual confounding (e.g. by maternal factors, SES)
Cave 2019a	L	Y	Y	CD	Y	N	Y	Y	N	N	Y	Y	CD	N	Y	Moderate	Potential measurement error by subjective carer-reported racism exposure (not validated)
Cave 2019b	L	Y	Y	CD	Y	N	Y	Y	Y	N	Y	Y	CD	N	Y	Moderate	Potential measurement error by subjective carer-reported racism exposure (not validated)
Shepherd 2017	L	Y	Y	CD	Y	N	Y	Y	N	N	Y	Y	CD	N	Y	Moderate	Potential measurement error by subjective carer-reported racism exposure (not validated)
Fatima 2020	L	Y	Y	CD	Y	N	Y	Y	Y	CD	N	Y	CD	Y	Y	Moderate	Potential for selection bias from LTFU/missing data; subjective carer-reported exposure; adjustment for potential confounders not decided prior to analysis
Haysom 2013	L/ CS	Y	Y	Y	Y	N	N	N	N	N	N	Y	CD	NA	Y	Moderate	Full list of variables adjusted for not stated
Singh 2003	L	Y	Y	Y	Y	N	Y	Y	Y	Y	N	Y	CD	N	N	Moderate	Potential for selection bias from missing data; potential for residual confounding (e.g. by birth/maternal factors, sex)
Mackerras 2003	CS	Y	Y	Y	Y	N	N	N	NA	Y	N	Y	CD	Y	Y	Moderate	Cross-sectional data; some potential confounding by earlier life remoteness; unadjusted measures reported but authors report adjusted measures were not materially different
Larkins 2017	CS	Y	Y	CD	Y	N	N	N	Y	N	N	Y	CD	NA	Y	Moderate	Cross-sectional data; potential for selection bias from missing/implausible data; some subjective carer-reported exposures, with potential for measurement error

Article ID	Design	Criteria number														Risk of bias	Main bias issues
		1	2	3	4	5	6	7	8	9	10	11	12	13	14		
Singh 2004	CS	Y	N	N	Y	N	N	N	Y	Y	N	Y	N	NA	N	Moderate	Cross-sectional data; potential for residual confounding (e.g. by birth/maternal factors, sex)
Valery 2012	CS	Y	Y	N	Y	Y	N	N	Y	N	N	Y	N	NA	Y	Moderate	Cross-sectional data; subjective self-reported exposures, with potential for differential recall bias (a known issue for dietary variables); potential selection bias by differential participation by island
Sjoholm 2020	L	Y	Y	Y	Y	N	Y	Y	Y	Y	Y	Y	CD	N	N	Moderate/ High	Unadjusted descriptive statistics (except regression analyses of BMI/WHtR tracking); potential for selection bias from LTFU/missing data (less risk for BMI/WHtR tracking where sensitivity analyses conducted)
Riley 2021	CS	Y	Y	CD	Y	N	N	N	Y	Y	N	N	CD	NA	N	Moderate/ High	Cross-sectional data; potential for selection bias from missing data; potential for confounding (BMI associations high risk); potential outcome misclassification (adult cutoffs)
Sevoyan 2019	CS	Y	Y	Y	N	N	N	N	Y	N	N	Y	CD	NA	Y	Moderate/ High	Cross-sectional data; unadjusted descriptive statistics for individual MetS components (high risk); potential for selection bias from LTFU/missing data
Juonala 2016	L	Y	Y	Y	Y	N	Y	Y	N	Y	N	Y	CD	N	N	High	Unadjusted descriptive statistics (measures included were secondary to main aim of study); potential for selection bias from LTFU/missing data
Sayers 2011	L	Y	Y	Y	Y	N	Y	Y	N	Y	NA	N	CD	N	N	High	Unadjusted descriptive statistics; potential outcome misclassification for <18 years; potential for selection bias from LTFU/missing data
Pringle 2019	L	Y	Y	CD	Y	N	Y	Y	Y	Y	NA	Y	CD	Y	N	High	Potential for selection bias from LTFU/missing data; small sample size

Article ID	Design	Criteria number														Risk of bias	Main bias issues
		1	2	3	4	5	6	7	8	9	10	11	12	13	14		
																	issues; some potential confounders not adjusted for
Denney-Wilson 2020	L	Y	Y	Y	N	N	Y	Y	N	Y	NA	Y	CD	N	N	High	Potential for selection bias from LTFU/missing data; adjustment for potential confounders not decided prior to analysis, with some inappropriate adjustment (adjustment for factors on causal pathway); small sample size issues
Webster 2013	L	Y	Y	Y	Y	N	Y	Y	N	Y	NA	Y	CD	N	N	High	Potential for selection bias from LTFU/missing data; no potential confounders not adjusted for
Campbell 2019	L	Y	Y	Y	Y	Y	Y	Y	Y	N	N	CD	Y	N	N	High	Unadjusted measures reported, potential for confounding; potential for selection bias from missing data, selection on clinic attendance; potential for measurement error from missing retrospective data (e.g. care received at other health services)
Braun 1996	L/ CS	Y	Y	N	Y	N	Y	Y	Y	Y	N	Y	CD	N	N	High	Mostly cross-sectional data; unadjusted descriptive statistics; potential for selection bias from LTFU
Thurber 2013	L/ CS	Y	Y	CD	Y	N	N	N	Y	Y	N	Y	CD	N	N	High	Unadjusted descriptive statistics; potential for selection bias from LTFU/missing data
Sellers 2008	CS	Y	Y	Y	Y	N	N	N	Y	Y	N	Y	CD	Y	N	High	Cross-sectional data; unadjusted for potential confounding
Priest 2011	CS	Y	Y	Y	Y	N	N	N	N	N	N	Y	CD	N	N	High	Cross-sectional data; unadjusted univariate association; subjective self-reported exposure (not validated); potential for selection bias from LTFU/missing data
Deacon-Crouch 2018	CS	Y	Y	CD	Y	N	N	N	Y	N	N	Y	CD	N	N	High	Cross-sectional data; subjective carer-reported exposure, with potential for differential recall bias; potential for selection bias from missing data; main

Article ID	Design	Criteria number														Risk of bias	Main bias issues
		1	2	3	4	5	6	7	8	9	10	11	12	13	14		
																	results reported are unadjusted measures, unclear how to interpret adjusted measure of association (exposure and outcome variables used in adjusted analysis not stated); complex sampling design not accounted for in analyses (e.g. clustering)
Esler 2016	CS	Y	Y	Y	Y	N	N	N	N	Y	N	Y	N	NA	N	High	Cross-sectional data; potential for residual confounding high for BMI, TG associations (behavioral factors, SES); adjustment for potential confounders not decided prior to analysis, with some inappropriate adjustment (adjustment for factors on causal pathway e.g. for sex); potential for outcome misclassification for participants with one BP measure
Smith 1992	CS	Y	Y	Y	Y	Y	N	N	Y	Y	N	Y	N	NA	N	High	Cross-sectional data; potential for residual confounding (e.g. by behavioral factors, SES); subjective self-report exposures, with potential for recall bias
Daniel 2002	CS	Y	Y	Y	N	N	N	N	Y	Y	N	Y	N	NA	N	High	Cross-sectional data; potential for residual confounding of association for age subgroup included (e.g. by age, behavioral factors, SES)
Schutte 2005	CS	Y	N	Y	CD	N	N	N	Y	Y	N	Y	N	NA	N	High	Cross-sectional data; potential for residual confounding of association for age subgroup included (e.g. by age, behavioral factors, SES)
Spurrier 2012	CS	Y	Y	Y	Y	N	N	N	Y	Y	N	Y	Y	NA	N	High	Cross-sectional data; unadjusted descriptive statistics; potential selection bias through exclusion of remote communities
Valery 2009	CS	Y	Y	Y	Y	N	N	N	N	Y	N	N	N	NA	N	High	Cross-sectional data; unadjusted descriptive statistics (main aim to measure

Article ID	Design	Criteria number														Risk of bias	Main bias issues
		1	2	3	4	5	6	7	8	9	10	11	12	13	14		
																	prevalence); potential outcome misclassification for <12 years participants
Angelino 2017	Mixed methods (CS)	Y	Y	N	Y	Y	N	N	N	N	N	Y	N	NA	N	High	Not peer reviewed; cross-sectional data; missing key data to interpret associations (group sizes, denominators, missing data); exposures not measured/defined consistently; severe risk of selection bias, confounding and measurement error

CD = cannot determine; CS = cross-sectional; L = longitudinal; LTFU = loss to follow-up; N = No; NA = Not applicable; Y = Yes

Table S5. Results from the NHLBI quality assessment tool for controlled intervention studies.

Article	Design	Criteria number														Risk of bias	Main bias issues
		1	2	3	4	5	6	7	8	9	10	11	12	13	14		
Smithers 2021	RCT	Y	Y	Y	N	Y	Y	N	Y	Y	CD	Y	CD	Y	Y	Low	Bias acknowledged/managed
Smithers 2017	RCT	Y	Y	Y	N	Y	Y	N	Y	Y	CD	Y	CD	Y	Y	Moderate	Potential for selection bias from LTFU/missing data

CD = cannot determine; LTFU = loss to follow-up; N = No; RCT = randomized controlled trial; Y = Yes

Table S6. Results from the NHLBI quality assessment tool for before-after (pre-post) studies.

Article	Criteria number												Risk of bias	Main bias issues
	1	2	3	4	5	6	7	8	9	10	11	12		
Gwynn 2014	Y	Y	Y	N	N	N	Y	N	NA	Y	N	Y	High	Analysis methods do not account for study design bias
Black 2013	Y	Y	N	Y	N	Y	Y	N	Y	Y	N	Y	High	Analysis methods do not account for study design bias

N = No; NA = Not applicable; Y = Yes

Table S7. Aboriginal and Torres Strait Islander Quality Appraisal Tool results.

Study	Question number														SCORE
	1	2	3	4	5	6	7	8	9	10	11	12	13	14	
MRDPP	Y	Y	Y	Y	Y	Y	Y	Y	P	N	Y	Y	Y	Y	12.5
LSIC	Y	Y	Y	Y	Y	U	U	Y	Y	Y	Y	Y	Y	Y	12
SEARCH	Y	Y	Y	Y	Y	U	U	Y	Y	Y	Y	Y	Y	Y	12
Schutte 2005, Daniel 2002	Y	Y	Y	Y	Y	U	U	Y	N	N	Y	Y	Y	Y	10
Gudaga	Y	Y	Y	Y	Y	U	U	U	N	N	Y	Y	Y	Y	9
Baby Teeth Talk	Y	Y	Y	Y	P	U	U	U	P	N	P	Y	Y	Y	8.5
Gomeroi gaaynggal	Y	Y	Y	Y	Y	U	U	U	N	N	U	Y	Y	Y	8
Esler 2016	Y	Y	P	Y	Y	U	U	U	N	N	Y	P	P	Y	7.5
ABC	U	Y	U	Y	Y	U	U	U	N	N	Y	Y	Y	Y	7
Angelino 2017	U	Y	Y	Y	Y	U	U	Y	N	N	P	P	U	Y	7
Campbell 2019	P	Y	Y	Y	Y	U	U	Y	N	N	U	U	U	U	5.5
Black 2013	Y	Y	U	Y	U	U	U	U	P	N	U	Y	U	P	5
Singh 2003, Singh 2004	U	Y	P	Y	U	U	U	U	N	N	U	U	P	U	3
Valery 2009, Valery 2012	U	P	Y	P	P	U	U	U	N	N	U	U	U	U	2.5
Spurrier 2012	U	U	Y	P	U	U	U	U	N	N	U	U	U	U	1.5
Braun 1996	U	Y	U	U	U	U	U	U	N	N	U	U	U	U	1
Haysom 2013	U	U	U	U	U	U	U	U	N	N	Y	U	U	U	1
Smith 1992	U	U	U	U	U	U	U	U	N	N	U	U	U	U	0

N = No; P = Partially; U = Unclear; Y = Yes
